# Bridging the lung cancer screening eligibility gap: evaluation of guideline applicability in asymptomatic patients^☆^

**DOI:** 10.1016/j.mmr.2026.100020

**Published:** 2026-04-15

**Authors:** Chen-Ran Wang, Ren-Da Li, Pan Wang, Xue-Si Dong, Hao Zhang, Qing-Peng Zeng, Jia-Xin Xie, Zi-Lin Luo, Xiao-Lu Chen, Ya-Di Zheng, Ji-Bin Li, Yong-Jie Xu, Fei Wang, Ni Li, Feng-Wei Tan, Jie He

**Affiliations:** a Office of Cancer Screening, National Cancer Center/National Clinical Research Center for Cancer/Cancer Hospital, Chinese Academy of Medical Sciences and Peking Union Medical College, Beijing 100021, China; b Chinese Academy of Medical Sciences Key Laboratory for National Cancer Big Data Analysis and Implement, Chinese Academy of Medical Sciences and Peking Union Medical College, Beijing 100021, China; c Department of Thoracic Surgery, National Cancer Center/National Clinical Research Center for Cancer/Cancer Hospital, Chinese Academy of Medical Sciences and Peking Union Medical College, Beijing 100021, China

**Keywords:** Lung cancer, Screening eligibility, US Preventive Services Task Force criteria, Real-world evidence

## Abstract

**Background:**

Early-onset (<50 years) and never-smoker lung cancers are increasing global concerns. The emerging trends challenge current screening guidelines, which focus on adults aged >50 years with heavy smoking histories. We applied the US Preventive Services Task Force (USPSTF) 2021 screening criteria as the primary definition of high-risk individuals eligible for lung cancer screening and assessed the potential optimization of these criteria using real-world lung cancer data in China.

**Methods:**

In this nationwide, multicenter, hospital-based observational study, we enrolled asymptomatic patients with surgically resected primary lung cancer across 26 tertiary hospitals from January 1, 2014 to December 31, 2021. Screening eligibility was classified using USPSTF 2021 criteria (aged 50−80 years, ≥20 pack-year smoking history, and ≤15 quit-years for former smokers). Temporal trends in eligibility, screening utilization, and mortality risks were assessed through joinpoint regression and Cox proportional hazards models.

**Results:**

A total of 106,266 asymptomatic patients with lung cancer were enrolled. Among the 102,555 patients with complete age and smoking information, only 8.8% (8985/102,555) met the USPSTF 2021 eligibility criteria. The eligibility proportion declined sharply from 21.6% (350/1617) in 2014 to 6.1% (1737/28,582) in 2021, with the annual percentage change being −17.4% [95% confidence interval (CI) −19.1 to −15.9]. Patients with screening utilization, irrespective of eligibility status, demonstrated a higher proportion of stage Ia diagnoses compared with those who were not screened. Screening- ineligible group exhibited 40% lower mortality risk overall [adjusted hazard ratio (*HR*)=0.60, 95% CI 0.55−0.66], with consistent survival advantages across stage I (adjusted *HR*=0.63, 95% CI 0.54−0.74) and stage III (adjusted *HR*=0.76, 95% CI 0.64−0.90) subgroups.

**Conclusions:**

Rigid age- and smoking-based criteria overlook substantial at-risk populations in China. Implementing individualized risk stratification is essential to advance equitable lung cancer screening.

## 1 Background

Lung cancer accounted for 12.4% of all newly diagnosed cancer cases and 18.7% of all cancer-related deaths worldwide in 2022 [1]. In China, lung cancer is the top cancer type of cancer-associated incidence and mortality, representing 22.0% of new cancer cases and 28.5% of total cancer deaths, respectively [2]. While traditionally considered a disease of heavy smokers aged ≥50 years, emerging epidemiological patterns reveal a concerning rise in early-onset cases (aged <50 years) and never-smoker lung cancers, particularly across East Asia [3], [4]. Early-onset lung cancer is characterized by a high proportion of never-smokers and lung adenocarcinoma [5], and it boasts a higher survival rate compared with late-onset cancer [6]. Additionally, in comparison to ever-smokers with lung cancer, non-smoking patients are more likely to be females [7], exhibit adenocarcinoma histology [7], and have a more favorable prognosis [8]. These shifting demographics underscore the urgent need for optimized screening approaches targeting non-traditional high-risk populations.

Low-dose CT (LDCT) screening has been shown in RCT trials to significantly reduce lung cancer mortality [9], [10]. Practical evidence from population-based screening programs further supports the feasibility of expanding screening eligibility in China [11], [12]. However, the standard categorical eligibility criteria, which rely solely on age and smoking history to determine eligibility, are overly simplistic and insufficient. For example, the 2021 US Preventive Services Task Force (USPSTF) recommendation advises annual LDCT screening for asymptomatic adults aged 50−80 years with a smoking history of ≥20 pack-years, who currently smoke or have quit within the past 15 years [13]. Although this update improves upon the 2013 USPSTF guidelines by lowering the age threshold from 55 to 50 years and the smoking threshold from 30 to 20 pack-years, the criteria remain rigid and one-size-fits-all [14]. As a result, a considerable proportion of lung cancer cases may be missed, particularly among individuals under 50 (or 55) years of age, those with less than 20 (or 30) pack-years of smoking, or never-smokers [13], [14]. Similarly, the high-risk definition outlined in the China Guideline for the Screening and Early Detection of Lung Cancer limits eligibility to individuals aged 50−74 years with a smoking history of at least 30 pack-years [15]. This definition does not adequately reflect the unique epidemiological profile of lung cancer in China, where a considerable burden exists among younger adults (aged <50 years) and never-smokers [15]. Broadening screening criteria to include these populations may yield clinically meaningful gains in early detection and improved survival.

Previous studies used screening cohort or national survey data to assess populations meeting UPSTF criteria [16], [17], [18], [19], [20], [21], [22]. While these studies have identified disparities in screening eligibility, most have focused on differences by sex [16], race, or ethnicity [17], [18], and were often constrained by small sample sizes and time frames [19], [20]. In the United States, only 5% to 15% of individuals meeting USPSTF criteria undergo screening [21], [22]. However, the evolution of LDCT screening eligibility in China over time remains poorly understood, largely due to the lack of comprehensive, individual-level nationwide data. Using a large-scale, nationally representative, real-world dataset, this study aims to: 1) assess the current landscape and temporal trends of screening eligibility based on composite and individual USPSTF criteria; 2) quantify screening utilization stratified by eligibility status and examine their association with lung cancer outcomes. In the context of an ongoing revolution in predictive, personalized, preventive, and participatory (P4) cancer medicine [23], this study supports the need for equitable, data-driven, and context-specific screening strategies adapted to the Chinese population. In particular, by incorporating the core principle of personalization into a proactive approach for lung cancer screening, the findings of this study may guide more accurate risk stratification and targeted screening invitations, ultimately informing the development of more inclusive and context-sensitive screening policies.

## 2 Methods

### 2.1 Study design and data source

This study has been reported in accordance with the Strengthening the Reporting of Observational Studies in Epidemiology (STROBE) statement [24]. It is registered with ClinicalTrials.gov (NCT06255197) and is part of the China National Cancer Center (NCC) LungReal Study, a nationwide, multicenter, hospital-based observational study designed to investigate the clinical characteristics, treatment patterns, and outcomes of patients with surgically resected lung cancer. The LungReal Study included patients who were newly diagnosed with primary lung cancer and underwent curative-intent surgery from 26 tertiary hospitals across China between January 1, 2014, and December 31, 2021. A detailed description of this real-world dataset is publicly available on the website of the National Library of Medicine (https://www.clinicaltrials.gov/study/NCT06255197?cond=NCT06255197&rank=1) [25]. Detailed proposal of the LungReal Study is given in Additional file 1.

In addition to the main clinical analysis of the LungReal Study dataset, we performed a complementary analysis using data from the China National Lung Cancer Screening (NLCS) programme, a multicenter, population-based, prospective cohort, which further supports the robustness of the study findings. Details of the NLCS study design have been published previously [11], [26]. We selected 5 (Shenyang, Beijing, Hangzhou, Hefei, and Ningbo) of the 12 participating cities (the remaining cities were Changsha, Zhengzhou, Zhumadian, Anyang, Quzhou, Xuzhou, and Nanjing) as the complementary analysis dataset, based on the inclusion of their local cancer registries in the Cancer Incidence in Five Continents (CI5) database [27].

### 2.2 Epidemiological and clinical indicators

#### 2.2.1 Epidemiological indicators

To address the objective of this study, we analyzed demographic and health-related variables from the LungReal Study, including age at diagnosis, sex, smoking status (classified as current smoker, former smoker, or never smoker), duration of smoking, number of cigarette packs smoked per day, years since smoking cessation (for former smokers), family history of lung cancer in first-degree relatives, presence of comorbidity, and medical insurance status. Age, sex, and medical insurance status were obtained from the front page of the electronic health record (EHR). Information on smoking status, smoking duration, cigarette consumption, years since smoking cessation, family history of lung cancer, and comorbidities was extracted from the patient-reported present history documented in admission records within the EHR. Smokers were defined as individuals who currently smoked or had previously smoked at least one cigarette per day for more than 6 months [28]. Smoking pack-year was calculated by multiplying the number of cigarette packs smoked per day (one pack=20 cigarettes) by the total number of years individual had smoked [28]. For former smokers, the number of years since quitting was also recorded. Never smokers were defined as individuals with no history of active tobacco use [29]. Comorbidity was defined as the presence of 1 or more chronic health conditions or functional impairments [30]. For the purpose of this study, comorbidity included at least 1 of the following diagnoses: chronic respiratory diseases, hypertension, diabetes, or coronary heart disease. Specific diagnostic codes based on the international classification of diseases, tenth revision (ICD-10) are listed in Additional file 2: Table S1 [31]. Information on the hospital region (eastern, central, and western) and the surgical volume of each hospital was also reported.

Epidemiological variables from the NLCS dataset, including demographic characteristics, smoking status, family history of lung cancer, and comorbidities were collected using structured questionnaires at cohort entry. Detailed information is shown in the previous publications [11].

#### 2.2.2 Clinical indicators

Clinical indicators assessed in the LungReal Study included histology type of lung cancer, stage at diagnosis, and overall survival time. Lung cancer cases were identified using diagnosis information and/or ICD codes including the ninth version (ICD-9) [32], ICD-10 [31], and the ICD for oncology (ICD-O) topography codes [33], obtained from the front page, admission records and/or pathology reports of EHR. Stage at diagnosis were extracted from the front page and admission records of EHR. Tumor staging was determined according to the 8th edition of the tumor-node-metastasis (TNM) classification system, as defined by the American Joint Committee on Cancer [34]. The primary endpoint was overall survival (OS), which was defined as the time from the date of surgery (the study entry point) to the date of death from any cause or the date of administrative censoring (December 31, 2021), whichever occurred first. Survival data were obtained through harmonization of re-visit or readmission records from participating centers, active follow-up conducted by these centers, and a linkage to the National Cancer Data Linkage (NCDL) Platform of China, which was established under a cooperative framework between NCC and the Chinese Center for Disease Control and Prevention. The procedures of obtaining clinical indicators and follow-up data in detail are provided in Additional file 1.

Clinical indicators assessed in the NLCS study were lung cancer diagnosis and stage. Lung cancer was coded as C34 according to ICD-10 [31]. Outcome data were retrieved from national linkages, including the cancer registry system and death surveillance system [11].

### 2.3 Identification of screening eligibility

Cancer screening is intended to identify malignant lesions during their asymptomatic preclinical stage [35]. Accordingly, only patients who were asymptomatic and had a histologically confirmed diagnosis of lung malignancy were considered eligible for inclusion in this study. In analyses based on the LungReal database, asymptomatic patients were defined as those who denied all lung cancer-related symptoms including cough, sputum production, blood in sputum, hemoptysis, shortness of breath, chest pain, hoarseness, and weight loss in their hospital admission records for lung malignancy [35]. These patients were mainly discovered by routine health examinations or opportunistic screening encounters. We excluded patients with diagnoses of pulmonary metastatic lesions, pulmonary lymphomas, or invasive carcinomas of mixed histology. In analyses of the NLSC cohort, incident lung cancer cases were those diagnosed during the follow-up period, with all participants being asymptomatic at baseline and eligible for inclusion [11].

Lung cancer patients were classified by screening eligibility based on the USPSTF and National Comprehensive Cancer Network (NCCN) guidelines, which are widely recognized and applied in current screening practice [13], [14], [36]. The primary eligibility definition followed the USPSTF 2021 guideline [13]. According to the USPSTF 2021 recommendation [13], LDCT screening eligibility is advised for asymptomatic individuals at high risk who fulfill all of the following criteria: 1) age criteria: aged 50−80 years; 2) smoking pack-year criteria: a smoking history of ≥20 pack-years for current or former smokers; 3) quit-year criteria: cessation ≤15 years for former smokers. Additionally, we also considered the USPSTF 2013 recommendations and the NCCN 2022 guideline [14], [36]. The USPSTF 2013 criteria defined high-risk individuals as those who were: 1) aged 55−80 years (age criteria); 2) had a smoking history of ≥30 pack-years for current or former smokers (smoking pack-year criteria); 3) had quit smoking for ≤15 years for former-smokers (quit-year criteria) [14]. The NCCN 2022 guideline recommended LDCT screening for individuals who were 1) aged ≥50 years (age criteria) and 2) had a ≥20 pack-year smoking history (smoking pack-year criteria) [36]. These international lung cancer screening guidelines were strictly followed without modification. The flow diagram detailing patient inclusion and exclusion procedures is provided in Additional file 2: Fig. S1.

Asymptomatic lung cancer patients were stratified according to guideline compliance. When applying the USPSTF recommendations, focusing primarily on these criteria, patients were classified as: 1) screening-eligible if they met all 3 criteria (age, smoking pack-year, or quit-year criteria) or at least 1 of the 3 criteria, and 2) screening-ineligible if they did not meet any of the 3 criteria [37]. When applying the NCCN recommendations, only the overall criteria were considered: patients fulfilling both age and smoking pack-year criteria were classified as screening-eligible, whereas those failing to meet either criterion were considered screening-ineligible. The China Guideline for the Screening and Early Detection of Lung Cancer was not included in this study for 2 reasons. First, several of its high-risk criteria are not quantitatively defined [e.g., family history or chronic obstructive pulmonary disease (COPD) history without explicit thresholds] [15]. Second, the implementation of the Chinese guideline remains at an early stage, and the absence of a nationally standardized evaluation framework for screening eligibility further limits its applicability in the current analyses.

### 2.4 Definition of screening utilization

As medical records did not specify the indication for chest CT scans whether for screening, diagnostic, or therapeutic purposes, patients from the LungReal Study were classified as having undergone screening if a chest CT scan had been performed more than 6 months before surgery in this study [38]. We adopted this 6-month cutoff, assuming that imaging performed well before surgery likely reflected asymptomatic detection rather than symptom-driven evaluation [38]. Among those with available CT scan data, patients were further stratified into 4 groups based on screening eligibility and utilization status: 1) ineligible without screening, 2) ineligible with screening, 3) eligible without screening, and 4) eligible with screening.

### 2.5 Evaluation of data quality

Quality evaluation for the NCC LungReal Study is described in Additional file 1. Briefly, quality of processed data was verified through automated checks against human-curated gold-standard datasets, and by manual comparison of processed data with raw data by trained data managers. Stratified sampling was applied to ensure generalizability across all participating centers.

For the NLCS, data initially collected by participating institutions were transmitted to the coordinating center at NCC via the web-based management system of the National Cancer Prevention and Control Network for double checking and central review [11].

### 2.6 Statistical analysis

#### 2.6.1 Descriptive and trend analyses

To describe the epidemiological and clinical characteristics of the study participants, categorical variables were summarized as frequencies and percentages [*n* (%)], while continuous variables were reported as median with interquartile range (IQR) due to abnormal distribution. We used Chi-square tests to compare the distributions of categorical variables between groups, in both LungReal and NLCS datasets. Temporal trends in screening eligibility from 2014 to 2021 were assessed using the Cochran-Armitage trend test. Additionally, we employed Joinpoint regression model to calculate the annual percentage change (APC) for each segment and the average annual percentage change (AAPC) for the entire study period (2014−2021). All models allowed a maximum of 1 Joinpoint (up to 2 linear segments), given the 8-year observation period, in line with the recommendation of 1 Joinpoint for 7−11 years of data [39]. Sex and smoking status (current vs. former) were used as stratification variables to account for the marked sex-specific differences in smoking prevalence and lung cancer risk, as well as the impact of smoking cessation on screening eligibility [40], [41].

#### 2.6.2 Survival analysis

Overall, 5-year survival rates were estimated using the Kaplan-Meier method and compared using the log-rank test. To assess the relationship between screening eligibility and mortality, we constructed a multivariable Cox proportional hazards model to calculate hazard ratios (*HR*s) and 95% confidence intervals (CIs), adjusting for sex, comorbidity, family history of lung cancer in first-degree relatives, insurance coverage, and residence. Additionally, to quantify the impact of the updated USPSTF 2021 criteria on screening eligibility, we compared the proportion of individuals who met the revised criteria with those who were ineligible under the previous USPSTF 2013 guideline. The USPSTF 2013 guideline required a ≥30 pack-year smoking history for eligibility [14]. The main analyses described were conducted using the LungReal Study dataset, with no imputation applied.

#### 2.6.3 Sensitivity analyses

Sensitivity analyses were performed using 4 approaches. First, we examined temporal trends in screening eligibility based on the NCCN 2022 criteria to determine whether the observed patterns were consistent across different screening guidelines [36]. Second, to address missing data within this real-world database, we applied multiple imputation using a Markov chain Monte Carlo method to supplement key missing epidemiological variables, including age, sex, smoking pack-years, years since smoking cessation, and medical insurance status [42]. The Cox proportional hazards model was then reconstructed to examine the relationship between screening eligibility and mortality risk using the imputed dataset. Third, to validate the robustness of survival outcomes by screening eligibility, we performed Cox proportional hazards model using the screening-eligible but unscreened group as the reference, defining screening as chest CT >12 months before surgery, excluding patients with pre-existing chronic respiratory diseases. Fourth, to account for the potential impact of competing risks on cause-specific mortality estimates, we performed the Fine-Gray model, treating death from other causes as a competing risk for lung cancer mortality.

APC and AAPC were calculated using Joinpoint Regression Program (version 5.2; Surveillance Research Program, National Cancer Institute, USA). Other statistical analyses were performed with SAS 9.4 (SAS Institute Inc., Cary, NC, USA). Statistical test was two-sided, and the significance level of *α* was 0.05.

## 3 Results

### 3.1 Overall screening eligibility

#### 3.1.1 Epidemiological profiles

We included 106,266 asymptomatic patients diagnosed with lung cancer between January 1, 2014, and December 31, 2021. Among the 102,555 patients with complete age and smoking information, 8.8% (8985/102,555) met the USPSTF 2021 eligibility criteria for lung cancer screening. Screening-ineligible patients were younger [median age: 58 years (IQR 49−65) vs. 63 (IQR 58−68), *P*<0.001] and predominantly never-smokers [94.9% (88,162/92,902)], with 4.1% (3766/92,902) having a <20 pack-year smoking history. Compared with ineligible patients, those meeting screening criteria had a higher prevalence of comorbidity [39.1% (3514/8985) vs. 16.5% (15,444/93,570), *P*<0.001] and were more likely to have medical insurance [85.9% (6512/7581) vs. 66.5% (53,063/79,745), *P*<0.001] (Table 1). Applying the USPSTF 2013 criteria, 6.3% (6483/103,065) of patients qualified for screening (Additional file 2**:** Fig. S2). The proportion of lung cancer cases that fulfilled the USPSTF 2021 criteria, including all three rules, age, and smoking pack-years increased by 2.5% (8.8 vs. 6.3), 13.7% (77.0 vs. 63.3), and 2.1% (9.9 vs. 7.8), respectively, compared with the USPSTF 2013 criteria (Additional file 2**:** Table S2).

**Table 1:** Characteristics of asymptomatic patients stratified by screening eligibility per the USPSTF 2021 criteria.

**Characteristic**	**Overall asymptomatic patients (*****n*****=106,266)**	**USPSTF 2021 criteria**		
		**Screening- ineligible (*****n=*****93,570)**	**Screening- eligible (*****n=*****8985)**	***P*****-value**
**Age [years, median (IQR)]**	58 (50−66)	58 (49−65)	63 (58−68)	<0.001
**Age [years,*****n*****(%)]**				<0.001
<50	23,776 (22.5)	23,776 (25.5)	0 (0.0)	
50−80	81,422 (77.0)	68,822 (73.9)	8985 (100.0)	
>80	524 (0.5)	524 (0.6)	0 (0.0)	
**Smoking status and pack years [*****n*****(%)]**				<0.001
Non-smokers	88,162 (86.5)	88,162 (94.9)	0 (0.0)	
Smokers with <20 pack years	3766 (3.7)	3766 (4.1)	0 (0.0)	
Smokers with 20−30 pack years	2135 (2.1)	475 (0.5)	1641 (18.3)	
Smokers with >30 pack years	7921 (7.8)	499 (0.5)	7344 (81.7)	
**Smoking quit years for former smokers [years,*****n*****(%)]**				<0.001
≤15	763 (19.6)	1032 (57.5)	1798 (100.0)	
>15	3133 (80.4)	763 (42.5)	0 (0.0)	
**Sex [*****n*****(%)]**				<0.001
Male	43,423 (40.9)	31,760 (33.9)	8714 (97.0)	
Female	62,842 (59.1)	61,810 (66.1)	270 (3.0)	
**Residence [*****n*****(%)]**				<0.001
Eastern region	96,826 (91.1)	86,864 (92.8)	7711 (85.8)	
Central and western regions	9440 (8.9)	6706 (7.2)	1274 (14.2)	
**Family history of lung cancer in first-degree relatives [*****n*****(%)]**				<0.001
No	100,436 (94.5)	88,731 (94.8)	8141 (90.6)	
Yes	5830 (5.5)	4839 (5.2)	844 (9.4)	
**Comorbidity [*****n*****(%)]**				
**Any comorbidity**				<0.001
No	86,211 (81.1)	78,126 (83.5)	5471 (60.9)	
Yes	20,055 (18.9)	15,444 (16.5)	3514 (39.1)	
**Chronic respiratory diseases**				<0.001
No	104,910 (98.7)	92,577 (98.9)	8717 (97.0)	
Yes	1356 (1.3)	993 (1.1)	268 (3.0)	
**Hypertension**				<0.001
No	90,797 (85.4)	81,535 (87.1)	6407 (71.3)	
Yes	15,469 (14.6)	12,035 (12.9)	2578 (28.7)	
**Diabetes**				<0.001
No	99,742 (93.9)	88,653 (94.8)	7715 (85.9)	
Yes	6524 (6.1)	4917 (5.3)	1270 (14.1)	
**Coronary heart disease**				<0.001
No	103,612 (97.5)	91,632 (97.9)	8380 (93.3)	
Yes	2654 (2.5)	1938 (2.1)	605 (6.7)	
**Medical insurance status [*****n*****(%)]**				<0.001
No medical insurance	27,980 (31.0)	26,682 (33.5)	1069 (14.1)	
Have medical insurance	62,211 (69.0)	53,063 (66.5)	6512 (85.9)	

Screening-eligible: asymptomatic individuals who fulfill all of the following criteria: 1) age criteria: aged 50−80 years; 2) smoking pack-year criteria: a smoking history of ≥20 pack-years for current or former smokers; 3) quit-year criteria: cessation ≤15 years for former smokers. Subgroup totals may not sum to the overall total because of missing values in some variables in the real-world database. IQR. Interquartile range; USPSTF. US Preventive Services Task Force

**Fig. 1:**
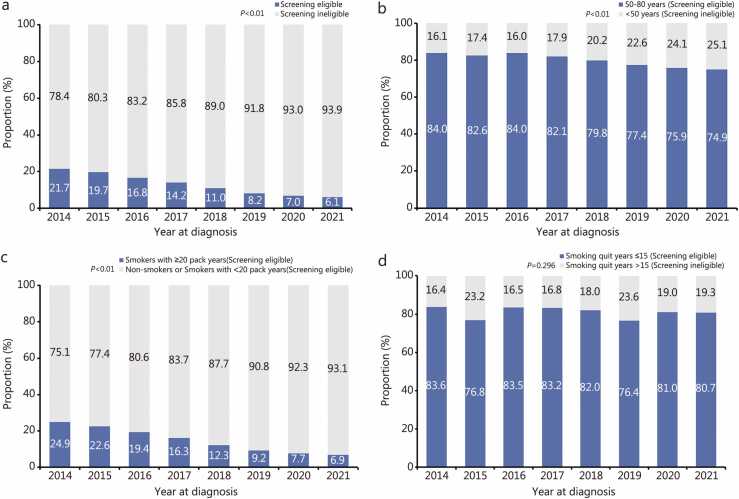
Trends of eligibility to lung cancer screening according to the US Preventive Services Task Force (USPSTF) 2021 criteria. **a** All USPSTF 2021 criteria. **b** Screening eligibility criteria on age. **c** Screening eligibility criteria on smoking pack years. **d** Screening eligibility criteria on smoking quit years for former smokers. Temporal trends in screening eligibility from 2014 to 2021 were assessed using the Cochran-Armitage trend test.

The eligible patients meeting the USPSTF 2021 criteria declined over time from 21.6% (350/1617) in 2014 to 6.1% (1737/28,582) in 2021 (*P* for trend <0.001) (Fig. 1**a**). Specifically, the proportion decreased from 84.0% (1475/1757) in 2014 to 74.9% (21,518/28,729) in 2021 (*P* for trend <0.001) (AAPC=−1.6%, 95% CI −1.9 to −1.4) for the age criteria (aged 50−80 years), from 24.9% (398/1600) to 6.9% (1956/28,518) for smoking criteria (*P* for trend <0.001) (AAPC=−18.2%, 95% CI −21.6 to −14.7), and from 83.6% (148/177) to 80.7% (498/617) for quit-year criteria (*P* for trend =0.296) (AAPC=−0.3%, 95% CI −1.5 to 0.9), respectively (Additional file 2**:** Table S3; Fig. 1**b-d**).

#### 3.1.2 Stratification by sex and smoking status

Of screening-ineligible patients, 66.1% (61,810/93,570) were female and 33.9% (31,760/93,570) were male. The leading cause of screening ineligibility was non-smoking, in both males [86.3% (27,014/31,288)] and females [99.2% (61,148/61,614)] (Additional file 2**:** Table S4). Screening eligibility declined sharply in both sexes (*P* for trend for both <0.001) (Additional file 2**:** Fig. S3), from 41.8% (335/801) in 2014 to 16.1% (1693/10,523) in 2021 in males (AAPC=−13.6%, 95% CI −16.0 to −11.6), and from 1.8% (15/816) to 0.2% (44/18,059) in females (AAPC=−28.8%, 95% CI −33.3 to −24.1) (Additional file 2**:** Table S3). Current smokers consistently exhibited a higher eligibility than former smokers (*P* for trend=0.028 for current smokers and 0.106 for former smokers) (Additional file 2**:** Fig. S4), with Joinpoint analysis showing no significant change over time for both current (AAPC=−0.7%, 95% CI −2.0 to 0.6) and former smokers (AAPC=−1.5%, 95% CI −4.5 to 1.7) (Additional file 2**:** Table S3).

#### 3.1.3 Other factors

From 2014 to 2021, the proportion of screening-eligible asymptomatic patients from eastern regions was higher than that from central and western regions (in 2014: 92.3% vs. 7.7%, *P*<0.001; in 2021: 89.8% vs. 10.2%, *P*<0.001). The proportion was also higher in high surgical-volume hospitals than in lower-volume hospitals (in 2014: 99.4% vs. 0.6%, *P*<0.001; in 2021: 77.1% vs. 22.9%, *P*<0.001) among screening-eligible asymptomatic patients (Additional file 2**:** Figs. S5-S6).

### 3.2 Screening utilization

Among 3700 asymptomatic lung cancer patients with recorded screening utilization, 290 (7.8%) met eligibility criteria. Screening utilization was higher in eastern than in central and western regions, both among eligible [82.1% (238/290) vs. 75.0% (1499/1998), *P=*0.009] and ineligible patients [90.5% (3087/3410) vs. 88.1% (17,664/20,054), *P*<0.001]. Ineligible non-screened patients were more likely to have medical insurance than those screened [82.8% (12,947/15,630) vs. 78.8% (2012/2554), *P*<0.001] (Table 2).

**Table 2:** Distribution of screening utilization stratified by screening eligibility per the USPSTF 2021 criteria.

**Characteristic**	**Screening-ineligible**			**Screening-eligible**		
	**Non screening (*****n*****=20,054)**	**Screening (*****n*****=3410)**	***P*****-value**	**Non screening (*****n*****=1998)**	**Screening (*****n*****=290)**	***P*****-value**
**Age [years, median (IQR)]**	57 (49, 65)	59 (51, 66)	<0.001	63 (57, 68)	63 (58, 68)	0.885
**Age [years,*****n*****(%)]**			<0.001			NA
<50	5129 (25.6)	728 (21.4)		NA	NA	
50−80	14,813 (73.9)	2652 (77.8)		1998 (100.0)	290 (100.0)	
>80	112 (0.6)	30 (0.9)		NA	NA	
**Smoking status [*****n*****(%)]**			0.001			0.764
Non smokers	18,693 (94.6)	3243 (95.9)		NA	NA	
Smokers with <20 pack years	856 (4.3)	123 (3.6)		NA	NA	
Smokers with 20−30 pack years	106 (0.5)	5 (0.2)		344 (17.2)	52 (17.9)	
Smokers with >30 pack years	116 (0.6)	10 (0.3)		1654 (82.8)	238 (82.1)	
**Smoking quit years for former smokers [years,*****n*****(%)]**			0.053			NA
≤15	190 (42.3)	25 (30.9)		396 (100.0)	118 (100.0)	
>15	259 (57.7)	56 (69.1)		NA	NA	
**Sex [*****n*****(%)]**			0.111			0.672
Male	6567 (32.8)	1164 (34.1)		1941 (97.2)	283 (97.6)	
Female	13,487 (67.3)	2246 (65.9)		57 (2.9)	7 (2.4)	
**Residence [*****n*****(%)]**			<0.001			0.009
Eastern regions	17,664 (88.1)	3087 (90.5)		1499 (75.0)	238 (82.1)	
Central and western regions	2390 (11.9)	323 (9.5)		499 (25.0)	52 (17.9)	
**Family history of lung cancer in first-degree relatives [*****n*****(%)]**			0.131			0.115
No	18,895 (94.2)	3235 (94.9)		1851 (92.6)	261 (90.0)	
Yes	1159 (5.8)	175 (5.1)		147 (7.4)	29 (10.0)	
**Comorbidity [*****n*****(%)]**						
**Any comorbidity**			0.371			0.137
No	16,227 (80.9)	2737 (80.3)		1342 (67.2)	182 (62.8)	
Yes	3827 (19.1)	673 (19.7)		656 (32.8)	108 (37.2)	
**Chronic respiratory diseases**			0.218			0.386
No	19,752 (98.5)	3368 (98.8)		1955 (97.9)	286 (98.6)	
Yes	302 (1.5)	42 (1.2)		43 (2.2)	4 (1.4)	
**Hypertension**			0.208			0.352
No	17,109 (85.3)	2881 (84.5)		1531 (76.6)	215 (74.1)	
Yes	2945 (14.7)	529 (15.5)		467 (23.4)	75 (25.9)	
**Diabetes**			0.292			0.446
No	18,882 (94.2)	3195 (93.7)		1754 (87.8)	250 (86.2)	
Yes	1172 (5.8)	215 (6.3)		244 (12.2)	40 (13.8)	
**Coronary heart disease**			0.053			0.444
No	19,549 (97.5)	3343 (98.0)		1849 (92.5)	272 (93.8)	
Yes	505 (2.5)	67 (2.0)		149 (7.5)	18 (6.2)	
**Medical insurance status [*****n*****(%)]**			<0.001			0.363
No medical insurance	2683 (17.2)	542 (21.2)		227 (14.4)	28 (12.1)	
Have medical insurance	12,947 (82.8)	2012 (78.8)		1355 (85.7)	203 (87.9)	

Screening-eligible: asymptomatic individuals who fulfill all of the following criteria: 1) age criteria: aged 50−80 years; 2) smoking pack-year criteria: a smoking history of ≥20 pack-years for current or former smokers; 3) quit-year criteria: cessation ≤15 years for former smokers. Subgroup totals may not sum to the overall total because of missing values in some variables in the real-world database. NA. Not available; USPSTF. US Preventive Services Task Force

### 3.3 Lung cancer diagnosis and mortality

#### 3.3.1 Pathological stage and type

**Fig. 2:**
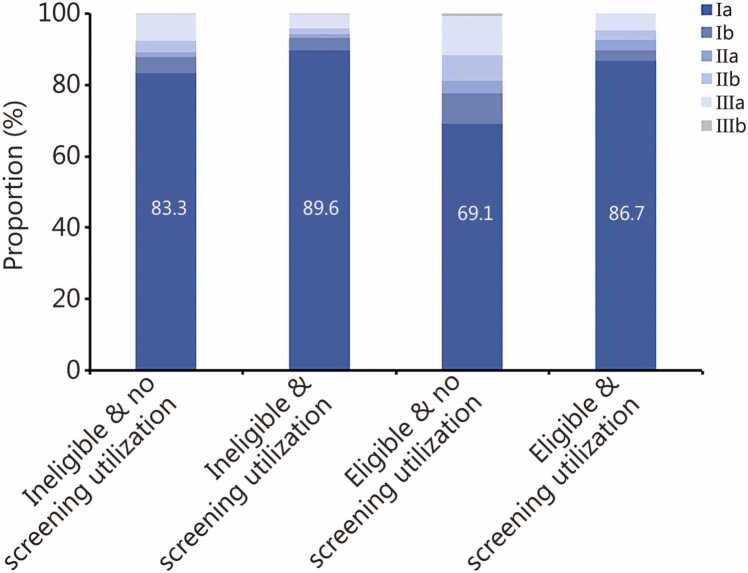
Stage at diagnosis and screening utilization stratified by screening eligibility according to the US Preventive Services Task Force (USPSTF) 2021 criteria.

Patients’ ineligible for screening under USPSTF 2021 criteria [73.7% (47,018/63,790)] were more likely to be diagnosed at stage Ia compared with those eligible [61.0% (4105/6731)] (Table 3). There was a higher proportion of stage Ia among the screened group compared to non-screened patients, both for eligible [86.7% (143/165) vs. 69.1% (1007/1458), *P*<0.001] or ineligible [89.6% (1569/1751) vs. 83.3% (11,698/14,036), *P*<0.001] patients (Fig. 2). Adenocarcinoma was more common in screening-ineligible patients [71.4% (57,240/80,143)] than in those screening-eligible [63.5% (4942/7787)]. The distribution of pathological type varied by age criteria: patients <50 years [59.9% (12,094/20,188)] had a lower proportion of adenocarcinoma than age-eligible patients [73.5% (51,550/70,150)]. Carcinoma in situ was more frequently diagnosed in screening-ineligible patients [9.0% (5722/63,790)] than in eligible ones [1.7% (111/6731)] (Table 3). Female patients’ ineligible for screening exhibited a higher proportion of stage Ia [76.3% (32,343/42,417) vs. 68.7% (14,675/21,373), *P*<0.001] and adenocarcinoma [72.5% (38,739/53,421) vs. 69.2% (18,501/26,722), *P*<0.001] than male counterparts, along with a lower mortality risk (Additional file 2**:** Table S4).

**Table 3:** Lung cancer diagnosis and mortality stratified by screening eligibility per the USPSTF 2021 criteria [*n* (%)].

**Lung cancer diagnosis and mortality**	**Overall asymptomatic patients**	**Screening eligibility**		**Age criteria**		**Pack-year criteria**		**Quit-year criteria**	
		**Screening- ineligible**	**Screening- eligible**	**Screening- ineligible (younger, <50 years)**	**Screening- eligible (≥50 and ≤80 years)**	**Screening- ineligible (Non-smokers or smokers with <20 pack years)**	**Screening- eligible (Smokers with ≥20 pack years)**	**Screening- ineligible (>15 years)**	**Screening- eligible (≤15 years)**
**Lung cancer diagnosis**									
**Stage at diagnosis**⁎									
Carcinoma in situ	6003 (8.2)	5722 (9.0)	111 (1.7)	2423 (15.8)	3523 (6.2)	5623 (9.0)	142 (1.9)	26 (4.3)	67 (2.8)
Ia	52,769 (72.1)	47,018 (73.7)	4105 (61.0)	11,209 (72.9)	41,075 (71.9)	46,258 (73.9)	4616 (61.4)	406 (67.0)	1582 (67.1)
Ib	3872 (5.3)	3052 (4.8)	585 (8.7)	323 (2.1)	3500 (6.1)	2976 (4.8)	642 (8.5)	48 (7.9)	174 (7.4)
IIa	1233 (1.7)	917 (1.4)	226 (3.4)	86 (0.6)	1134 (2.0)	892 (1.4)	244 (3.2)	20 (3.3)	60 (2.5)
IIb	3315 (4.5)	2385 (3.7)	757 (11.3)	398 (2.6)	2887 (5.1)	2293 (3.7)	824 (11.0)	43 (7.1)	205 (8.7)
IIIa	5592 (7.6)	4415 (6.9)	845 (12.6)	883 (5.7)	4654 (8.2)	4270 (6.8)	945 (12.6)	59 (9.7)	249 (10.6)
IIIb	387 (0.5)	269 (0.4)	100 (1.5)	47 (0.3)	338 (0.6)	258 (0.4)	105 (1.4)	4 (0.7)	21 (0.9)
IV	14 (0.0)	12 (0.0)	2 (0.0)	2 (0.0)	12 (0.0)	12 (0.0)	2 (0.0)	0 (0.0)	1 (0.0)
**Histological subtype**⁎									
Minimally invasive adenocarcinoma	12,901 (14.1)	12,450 (15.5)	281 (3.6)	5083 (25.2)	7766 (11.1)	12,313 (15.6)	337 (3.9)	40 (5.9)	111 (4.2)
Adenocarcinoma	64,385 (70.5)	57,240 (71.4)	4942 (63.5)	12,094 (59.9)	51,550 (73.5)	56,271 (71.5)	5614 (64.4)	522 (76.5)	1794 (67.6)
Squamous carcinoma	5234 (5.7)	2842 (3.6)	1808 (23.2)	239 (1.2)	4929 (7.0)	2715 (3.5)	1924 (22.1)	71 (10.4)	504 (19.0)
Other NSCLC	7947 (8.7)	7114 (8.9)	505 (6.5)	2717 (13.5)	5145 (7.3)	6957 (8.8)	579 (6.6)	46 (6.7)	193 (7.3)
Small-cell lung cancer	827 (0.9)	497 (0.6)	251 (3.2)	55 (0.3)	760 (1.1)	480 (0.6)	265 (3.0)	3 (0.4)	52 (2.0)
**Mortality**									
All-cause death	4693 (4.4)	3145 (3.4)	1146 (12.7)	481 (2.0)	4151 (5.1)	2979 (3.2)	1283 (12.8)	102 (13.4)	384 (12.3)
Alive or loss to follow-up	101,573 (95.6)	90,425 (96.6)	7839 (87.3)	23,295 (98.0)	77,271 (94.9)	88,949 (96.8)	8773 (87.2)	661 (86.6)	2749 (87.7)

⁎ Available data on stage at diagnosis and histological subtype were presented. Distributions of lung cancer diagnosis and mortality among asymptomatic patients aged >80 years were not shown due to limited sample size. There were no significant statistical differences (*P*>0.05) in distributions of stage at diagnosis and mortality between former-smokers with ≤15 quit years and those >15 quit years. Screening-eligible: asymptomatic individuals who fulfill all the following criteria: 1) age criteria: aged 50−80 years; 2) smoking pack-year criteria: a smoking history of ≥20 pack-years for current or former smokers; 3) quit-year criteria: cessation ≤15 years for former smokers. Subgroup totals may not sum to the overall total because of missing values in some variables in the real-world database. USPSTF. US Preventive Services Task Force; NSCLC. Non-small cell lung cancer

From 2014 to 2021, stage Ia diagnoses increased among screening-ineligible patients [66.4% (619/932) to 84.3% (12,347/14,642); AAPC=3.2%, 95% CI 1.6−4.6] (Additional file 2**:** Table S3 and Fig. S7a). This trend was consistent across ineligible subgroups. Among patients <50 years, stage Ia increased from 62.4% to 91.3% (AAPC=5.5%, 95% CI 3.7−7.0) (Additional file 2**:** Table S3 and Fig. S7b). Never-smokers and those with <20 pack-years also showed an increasing stage Ia detection [67.4% (595/883) to 84.4% (12,214/14,475); AAPC=3.0%, 95% CI 1.2−4.5] (Additional file 2**:** Table S3 and Fig. S7c), as did former smokers who had quit for >15 years [52.4% (11/21) to 76.9% (60/78); AAPC=5.1%, 95% CI 2.0−8.4] (Additional file 2**:** Table S3 and Fig. S7d).

#### 3.3.2 Mortality risk

**Fig. 3:**
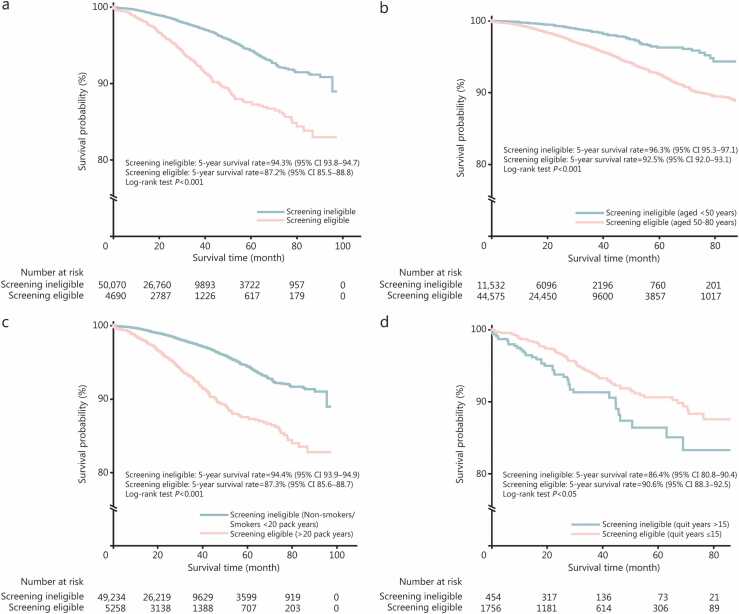
Kaplan-Meier estimates among asymptomatic lung cancer patients at stage I according to the US Preventive Services Task Force (USPSTF) 2021 criteria. **a** All USPSTF 2021 criteria. **b** Screening criteria on age. **c** Screening criteria on smoking pack years. **d** Screening criteria on smoking quit years for former smokers. Mortality cases for former smokers might be limited to compare steadily survival rates according to the screening eligibility criteria on smoking quit years. CI. Confidence interval.

**Fig. 4:**
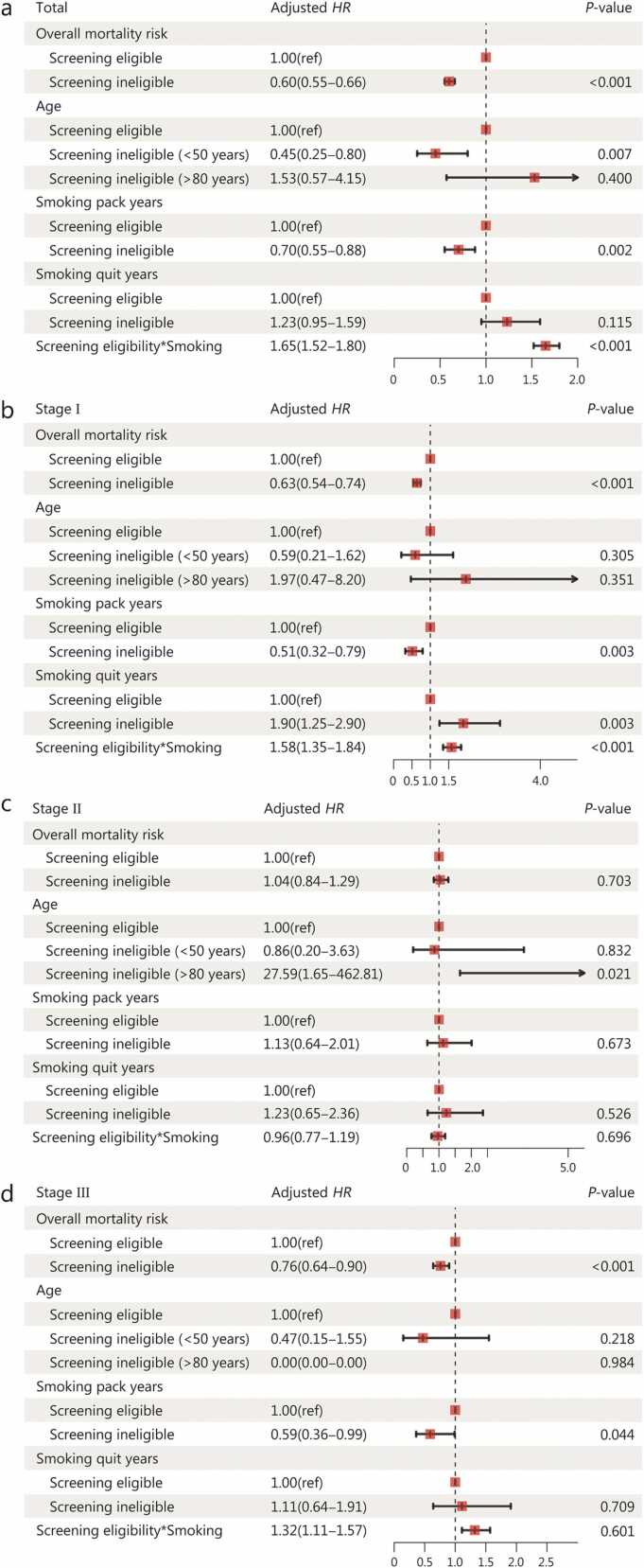
Mortality risk between asymptomatic patients eligible and ineligible to lung cancer screening according to the US Preventive Services Task Force (USPSTF) 2021 criteria. **a** Total number of asymptomatic patients. **b** Stage I. **c** Stage II. **d** Stage III. Hazard ratios (*HR*s) were adjusted for sex, comorbidity, family history of lung cancer in first-degree relatives, insurance coverage status, and residence. Adjusted *HR* for asymptomatic patients aged >80 years at stage III could not be estimated steadily due to the insufficient mortality cases. Interaction term represents screening eligibility (ineligible or eligible) and smoking status (non-smokers or smokers).

There were 1146 (12.7%, 1146/8985) all-cause deaths in the screening-eligible group and 3145 (3.4%, 3145/93,570) among ineligible patients (Table 3). Stage I patients who were screening-ineligible (94.3%, 95% CI 93.8−94.7) had a higher 5-year OS rate than those eligible (87.2%, 95% CI 85.5−88.8), with a pattern consistent across age [96.3% (95% CI 95.3−97.1) vs. 92.5% (95% CI 92.0−93.1)], smoking history [94.4% (95% CI 93.9−94.9) vs. 87.3% (95% CI 85.6−88.7)], and sex [Males: 91.1% (95% CI 90.0−92.1) vs. 86.9% (95% CI 85.0−88.5); Females: 95.7% (95% CI 95.2−96.2) vs. 88.1% (95% CI 69.4−95.7)] (Fig. 3; Additional file 2**:** Fig. S8). Overall, screening-ineligible patients had a lower risk of all-cause mortality (adjusted *HR*=0.60, 95% CI 0.55−0.66). Subset analyses indicated reduced mortality among screening-ineligible patients aged <50 years (adjusted *HR*=0.45, 95% CI 0.25−0.80), and non-smokers or smokers with a smoking history of <20 pack-years (adjusted *HR*=0.70, 95% CI 0.55−0.88]) compared with screening-eligible patients aged 50−80 years and those with a ≥20 pack-year smoking history, respectively (Fig. 4**a**). By stage, ineligible patients exhibited an overall lower risk of mortality at stage I (adjusted *HR*=0.63, 95% CI 0.54−0.74) and stage III (adjusted *HR*=0.76, 95% CI 0.64−0.90), compared with eligible patients (Fig. 4**b-d**). The significant interaction effects indicated that the association between screening eligibility and mortality risk differed across smoking status (Fig. 4**a-d**).

### 3.4 Sensitivity analyses

The proportion of asymptomatic patients eligible for lung cancer screening was slightly higher under NCCN 2022 criteria than USPSTF 2021 criteria [9.1% (9330/102,448) vs. 8.8% (8985/102,555)] (Additional file 2**:** Fig. S2). Temporal trends in the proportion of screening eligibility showed a consistent decrease across all guidelines (USPSTF 2021: from 21.7% to 6.1%; NCCN 2022: from 22.7% to 6.2%; USPSTF 2013: from 15.4% to 4.5%) (Additional file 2**:** Fig. S9). Mortality risk remained lower among ineligible patients compared with eligible individuals after imputing missing variables (Additional file 2**:** Table S5 and Fig. S10). Using eligible non-screened patients as the reference, decreased mortality risks persisted among both screening-ineligible patients overall (adjusted *HR*=0.62, 95% CI 0.49−0.77) and at stage I (adjusted *HR*=0.68, 95% CI 0.47−1.00) (Additional file 2**:** Table S6). These associations remained consistent when applying a screening definition of having chest CT over 12 months before surgery (patients overall: adjusted *HR*=0.63, 95% CI 0.51−0.79; at stage I: adjusted *HR*=0.69, 95% CI 0.48−1.00) (Additional file 2**:** Table S7), and excluding those with pre-existing chronic respiratory diseases (patients overall: adjusted *HR*=0.63, 95% CI 0.50−0.78; at stage I: adjusted *HR*=0.67, 95% CI 0.46−0.98) (Additional file 2**:** Table S8). Moreover, screening-ineligible patients showed a lower risk of lung cancer mortality (patients overall: adjusted *HR*=0.58, 95% CI 0.52−0.65; at stage I: adjusted *HR*=0.56, 95% CI 0.46−0.69) after accounting for competing risk (Additional file 2**:** Table S9).

### 3.5 Complementary analysis

Population-based complementary analysis showed that differences in epidemiological and clinical profiles between screening-eligible and -ineligible populations from 5 cities in the NLCS cohort were also broadly consistent with those observed in the main analysis. Under USPSTF 2021 criteria, patients who were ineligible for screening [68.0% (732/1076)] were more likely to be diagnosed at stage Ia compared with those were eligible [45.4% (143/315)]. When stratified by individual eligibility criteria, higher proportions of stage Ia diagnosis were consistently observed among patients who not meeting age criteria [83.2% (89/107)] and those not meeting pack-year criteria (68.3%) (725/1062), compared with their corresponding screening-eligible counterparts [61.2% (786/1284) and 45.6% (150/329), respectively] (Additional file 2**:** Tables. S10-S11).

## 4 Discussion

In this study, we evaluated the evolving characteristics and temporal trends in screening eligibility among asymptomatic patients with surgically resected lung cancer, using the updated 2021 USPSTF criteria. The findings revealed that a substantial and increasing proportion of asymptomatic lung cancer cases fell outside the current screening framework. Mortality comparisons between screening-eligible and ineligible groups suggest that individuals not meeting existing criteria may still benefit from early detection. These findings highlight the inadequacy of current screening criteria for identifying high-risk individuals in China.

The observed mismatch between eligibility and lung cancer occurrence underscores the limitations of a one-size-fits-all approach. Prior research has similarly demonstrated that the USPSTF criteria fail to account for sex [16], racial, or ethnic disparities in lung cancer risk [17], [18]. For example, analysis of the 2015 National Health Interview Survey showed higher eligibility rates among non-Hispanic White individuals compared with non-Hispanic Black, Asian, and Hispanic populations [17]. A recent study from the Southern Community Cohort Study and the Black Women’s Health Study further showed that using a ≥20-year smoking duration criterion, rather than a ≥20 pack-year standard, could improve eligibility equity and reduce racial disparities [43]. These findings collectively call for a shift toward more personalized, risk-based screening strategies that reflect population-specific risk profiles and promote equity in access to early detection.

We also identified age-specific disparities in screening eligibility, with patients aged <50 years notably underrepresented, underscoring the emerging public health challenge of early-onset lung cancer in China. The proportion of patients aged 21−40 years nearly tripled from 3.6% in 2012 to 9.0% in 2020 [44]; and national registry data indicated that lung cancer ranks among the 5 most common cancers among adults aged 20−49 years [45]. This study suggested that early-onset lung cancer cases exhibited distinct pathological and staging patterns compared with late-onset disease, potentially driven by evolving gene-environment interactions and increased exposure to modifiable risk factors such as tobacco use, elevated C-reactive protein levels, hypertension, and tea consumption [46]. Although China’s national screening programme which targets individuals aged 40−74 years is broader than international guidelines, previous research demonstrated that a one-off LDCT screening in individuals aged 40−54 years did not significantly reduce lung cancer or all-cause mortality [11]. Novel strategies incorporating multi-dimensional biomarkers (e.g., cell-free DNA fragmentomics [47], exhaled volatile organic compounds [48], and artificial intelligence-based risk modelling [49] hold promise for improving early detection in this growing at-risk demographic.

Moreover, the findings of this study revealed a rising proportion of lung cancer cases among never-smokers and light-smokers (<20 pack-years) who fall outside USPSTF screening criteria. Restricting screening eligibility to individuals with ≥20 pack-years may miss a substantial and growing subgroup [50]. Data from the Lung Cancer Database of West China Hospital similarly reported an increase in never-smoker cases from 41.3% in 2009 to 52.4% in 2018 [6]. A study further demonstrated that LDCT screening among never-smokers with additional risk factors, such as age over 60 years and family history of lung cancer, yielded a higher detection rate of invasive lung cancer [51]. These results reinforce the need for more individualized screening approaches that incorporate non-smoking-related risk factors, including family history [52], secondhand smoke exposure [29], and occupational hazards exposure [53]. However, in the absence of robust evidence on screening effectiveness in never-smokers and light-smokers, well-designed randomized controlled trials are urgently needed [54].

We identified persistent sex-based disparities in lung cancer screening eligibility, primarily attributable to differences in smoking prevalence. Data from the China Chronic Disease and Risk Factor Surveillance showed that while smoking rates among men declined from 58.4% in 2007 to 50.8% in 2018, rates among women remained extremely low, decreasing only marginally from 2.2% to 1.9% [40]. These trends parallel the sex-based screening eligibility gap observed in this study and highlight the limitations of applying uniform pack-year thresholds across sexes. The development of sex-specific lung cancer screening strategies is essential to improve risk stratification and ensure equitable access [55]. Importantly, we observed that geographic variation also influenced screening eligibility and utilization. Consistent with a previous study, patients attending larger hospitals in economically advantaged areas, particularly those with integrated lung health programmes, were more likely to receive opportunistic screening [56]. These institutions typically benefit from greater health care resources, including advanced imaging technologies and multidisciplinary teams capable of delivering high-quality LDCT interpretation [57]. Notably, the limitations of age- and smoking-based eligibility criteria are not unique to China and have also been observed in other countries [37], [58]. Despite differences in specific disparities, commonalities in health system structures suggest shared challenges. In the Chinese context, leveraging the national three-tiered healthcare system offers a pragmatic pathway to improve access to screening among underserved and high-risk populations.

The findings of this study show that a growing proportion of asymptomatic individuals ineligible for LDCT screening were nonetheless diagnosed with stage I lung cancer. Given the irreversible biological progression of lung cancer from precancerous lesions to carcinoma in situ, microinvasive disease, and ultimately invasive carcinoma, timely detection is essential [59]. Expanding eligibility to include high-risk individuals currently excluded by existing guidelines could enable earlier diagnosis and potentially prevent progression to advanced stages [60]. Moreover, we observed lower overall mortality among ineligible patients, including those diagnosed at stage I and stage III, suggesting that early detection and treatment may yield significant survival benefits even outside standard eligibility thresholds. Stratified analysis further revealed that never-smokers and individuals with <20 pack-years of smoking history had lower mortality risks. This may be partly explained by the higher prevalence of lung adenocarcinoma in never-smokers [61], a histological subtype more frequently diagnosed at early stages and associated with better prognosis [6], [62]. These findings support the need for risk-adapted screening approaches that move beyond smoking history alone. Evaluating the potential health benefits of screening among currently ineligible but high-risk populations is critical to improving the reach and equity of screening programmes.

However, a population-based cohort study raised concerns about overdiagnosis associated with expanding LDCT screening to lower-risk groups. This was evidenced by a marked increase in the incidence of early-stage lung cancer following the introduction of LDCT screening among non-smoking women, without a corresponding decline in late-stage disease [54]. Similarly, this analysis showed a higher proportion of carcinoma in situ among screening-ineligible patients, further suggesting the potential for overdiagnosis if screening is universally applied. In China, lung cancer screening is not currently reimbursed by the national health insurance system [63]. Prior studies have shown that insurance coverage is associated with higher screening uptake among high-risk men, whereas limited coverage remains a barrier [63], [64]. Nevertheless, expanding reimbursement raises concerns about equity and financial sustainability [65]. Hospital-based opportunistic screening for high-risk individuals outside current eligibility criteria may offer a more pragmatic approach. Beyond overdiagnosis, LDCT screening carries risks from downstream diagnostic procedures and associated complications [66]. It is therefore imperative to conduct rigorous risk-benefit evaluations in never-smokers and individuals with <20 pack-years of smoking exposure, to ensure that early detection confers meaningful benefit without unintended harm [67].

The findings do not support the exclusion of individuals who have quit smoking for more than 15 years from lung cancer screening. Under the USPSTF 2021 criteria, former smokers are less likely to be eligible for screening than current smokers due to the 15-year cessation threshold. This restriction may inadvertently incentivize individuals to resume smoking or misreport their smoking status to qualify for screening [36]. In this real-world study, those who quit >15 years showed no significant reduction in mortality (particularly among stage I cases), possibly due to older age at diagnosis. The findings align with the Iowa Women’s Health Study [68], and the Prostate, Lung, Colorectal, and Ovarian Cancer Screening Trial results [69]. Conversely, a recent meta-analysis suggests that ≥10 years of cessation improves survival [70]. The limited proportion of former smokers in this study (3.7%) reduced the statistical power to differentiate mortality risk by cessation duration (>15 vs. <15 years). More flexible risk-based criteria and the integration of novel biomarkers should be considered to better capture long-term risk among former smokers and inform optimized screening guidelines [71], [72].

This study also has several limitations. First, reliance on self-reported clinical symptoms and smoking history may introduce information bias, potentially leading to misclassification of screening eligibility and smoking status. Future studies incorporating standardized symptom assessments are needed to more accurately distinguish asymptomatic cases from those with atypical presentations. Moreover, selection biases such as Neyman’s bias (prevalence-incidence bias) and no-response bias may have potentially affected the findings. For example, lung cancer patients with comorbidities such as COPD may have modified their smoking behavior following diagnosis, complicating the accurate assessment of baseline smoking status. Second, all 26 participating hospitals were in urban areas, limiting the ability to examine urban-rural disparities in screening eligibility. Third, the lack of information on the clinical indication for CT scans precluded differentiation between diagnostic and screening use, possibly leading to overestimation of screening uptake in real-world practice. Fourth, as this cohort consisted solely of patients with surgically resected lung cancer, we were unable to account for the impact of adjuvant therapies on clinical outcomes, limiting direct mortality comparisons between screening-eligible and ineligible groups. Finally, although this large-scale, multicenter, hospital-based study provided valuable insights into screening patterns in China, the findings may not be fully generalizable to the broader population. However, this complementary analysis based on a prospective, population-based cohorts offer supportive evidence for the generalizability of these observations.

## 5 Conclusions

This study underscores the necessity of refining current guideline-based eligibility criteria to identify the risk profiles of individuals ultimately diagnosed with lung cancer more accurately. Considering the unique epidemiological characteristics of lung cancer in China, a risk-based personalized screening strategies that extend beyond age and smoking history should be developed to enhance the identification of high-risk individuals and promote more equitable access to screening.

## Abbreviations

AAPC: Average annual percentage change

APC: Annual percentage change

CI: Confidence interval

COPD: Chronic obstructive pulmonary disease

HR: Hazard ratio

LDCT: Low-dose computed tomography

NCC: National Cancer Center

NCCN: National Comprehensive Cancer Network

NLCS: China National Lung Cancer Screening

USPSTF: US Preventive Services Task Force

## Ethics approval and consent to participate

The LungReal Study is a retrospective, non-interventional study using de-identified information in all analyses. According to the guidelines of the Council of International Medical Science Organizations, informed consent could be waived with the approval of the Ethics Committee. Accordingly, a waiver of informed consent was obtained for this study. The LungReal Study was approved by the ethics committees of China National Cancer Center/Cancer Hospital, Chinese Academy of Medical Sciences and Peking Union Medical College (19/217-2001). The complementary analysis dataset from the China National Lung Cancer Screening (NLCS) programme was approved by the ethics committees of China National Cancer Center/Cancer Hospital, Chinese Academy of Medical Sciences and Peking Union Medical College (15-070/997).

## Funding

This study is funded by the Chinese Academy of Medical Sciences Clinical and Translational Medicine Research Programme (2022-I2M-C&T-B-060), the Chinese Academy of Medical Sciences Medical and Health Science and Technology Innovation Project (2021-I2M-1-061), the Chinese Academy of Medical Sciences for the Fundamental Research Funds for the Central Universities (2019PT320027), Noncommunicable Chronic Diseases-National Science and Technology Major Project (2024ZD0520000), National Natural Science Foundation of China (82273722; 82472994), and Central University Basic Research Fund of Peking Union Medical College (3332024216).

## Data Availability

The data and materials that support the findings of this study are available from the corresponding author upon reasonable request.

## Footnotes

Appendix A Supplementary data associated with this article can be found in the online version at doi:10.1016/j.mmr.2026.100020.
