# Supplementary material for: Bridging the lung cancer screening eligibility gap: evaluation of guideline applicability in asymptomatic patients
Source: Mil Med Res. 2026 Apr 15;13(1):100020. doi: 10.1016/j.mmr.2026.100020 (PMC13127149; doi:10.1016/j.mmr.2026.100020)
Supplement: Supplementary file 1 — Supplementary material [file mmc1.pdf]

Protocol for phase 1 study of characteristics, treatment patterns, and outcomes for patients with surgically resected lung cancers in China

(National Cancer Center LungReal Study)

Version 1.7

Version Date 2022-04-01

1 Introduction

According to the latest global cancer statistics from 2018, lung cancer ranks first among all malignancies in both incidence and mortality. Lung cancer is also the leading cancer type in China. Histologically, lung cancer is primarily classified into two major subtypes: non-small cell lung cancer (NSCLC) and small cell lung cancer (SCLC). NSCLC represents the most common form, constituting approximately 80%–85% of all lung cancer cases.

Advances in lung cancer screening among high-risk populations have led to a steady increase in the detection of early-stage disease. For patients with stage I–IIIa NSCLC, surgical resection remains the cornerstone of treatment and the primary therapeutic strategy. Moreover, the role of adjuvant chemotherapy has been firmly established; platinum-based regimens are now considered standard adjuvant therapy for patients with completely resected stage II–IIIa NSCLC. Despite significant improvements in survival outcomes conferred by surgery and adjuvant chemotherapy, the 5-year overall survival rate remains suboptimal at approximately 40%.

Consequently, considerable clinical research efforts are currently focused on identifying additional therapeutic approaches to reduce recurrence risk and further improve long-term survival. In patients harboring activating epidermal growth factor receptor (EGFR) mutations, postoperative adjuvant therapy with EGFR tyrosine kinase inhibitors (TKIs) has been actively investigated. The BR.19 and RADIANT trials evaluated adjuvant TKI therapy in unselected populations with stage Ib–IIIa NSCLC but failed to demonstrate a significant improvement in disease-free survival (DFS) compared with placebo. However, results from the CTONG1104 trial (published in 2018) and the EVAN study (reported at the 2017 World Conference on Lung Cancer) demonstrated that, in patients

with completely resected EGFR-mutant NSCLC, adjuvant EGFR-TKI therapy conferred a statistically significant DFS benefit over conventional chemotherapy.

For patients with stage IIIa NSCLC, definitive concurrent chemoradiotherapy remains the standard of care for those deemed unresectable. In potentially resectable cases, neoadjuvant therapy followed by surgery is an accepted alternative; however, the optimal neoadjuvant regimen, whether chemotherapy alone, sequential chemoradiotherapy, concurrent chemoradiotherapy, or chemotherapy followed by concurrent chemoradiotherapy, remains to be definitively established. The advent of targeted therapies and immune checkpoint inhibitors has further expanded the landscape of neoadjuvant strategies. In 2018, the European Society for Medical Oncology (ESMO) reported findings from CTONG1103, the first global trial of neoadjuvant targeted therapy, which showed superior efficacy of neoadjuvant EGFR-TKIs over neoadjuvant chemotherapy in patients with resectable EGFR-mutant stage IIIA-N2 NSCLC. Meanwhile, trials such as NEOSTAR, NADIM, and LCMC3 have explored the use of programmed death-1 (PD-1) inhibitors in the neoadjuvant setting for resectable NSCLC, offering promising preliminary evidence for the potential of neoadjuvant immunotherapy.

NSCLC remains one of the most intensively researched areas in oncology, yet numerous critical questions persist. Surgical approaches for lung cancer are diverse and continually evolving, raising important questions regarding optimal technique selection and the extent of lymph node dissection, particularly in early-stage disease. Retrospective data suggest that, for clinical stage I NSCLC, thoracoscopic and robot-assisted lobectomy with lymphadenectomy yield oncologic outcomes comparable to those of open thoracotomy. However, whether selective, limited lymph node dissection is appropriate in select early-stage patients requires further prospective validation. Beyond

surgical technique itself, integrating surgery with systemic therapies to optimize long-term survival has become a central focus of international research. Key unresolved issues include the optimal neoadjuvant strategy, the role and sequencing of postoperative radiotherapy and chemotherapy in stages IIb–IIIa, and the impact of adjuvant targeted or immunotherapies on long-term survival and patterns of recurrence.

Traditional clinical research, exemplified by randomized controlled trials (RCTs), provides high internal validity through tightly controlled conditions and homogeneous patient populations. However, the strict eligibility criteria and simplified intervention protocols of RCTs often limit their generalizability to real-world clinical practice, thereby constraining their utility in guiding personalized medicine. In contrast, real-world evidence (RWE), derived from routine clinical settings, offers high external validity, greater flexibility in study design, and enhanced feasibility, making it increasingly valuable to researchers. Recent advances in health informatics and big-data analytics in oncology have led to exponential growth in the availability of high-quality RWE, enabling robust real-world studies that complement traditional trial data.

Numerous real-world studies on lung cancer have already been conducted, with large-scale analyses based on databases such as the National Cancer Database (NCDB) and the Surveillance, Epidemiology, and End Results (SEER) program gaining prominence. For instance, investigators from Weill Cornell Medicine used SEER data to assess the prognostic impact of lymph node dissection extent in patients with NSCLC tumors ≤2 cm and pathologically node-negative disease, suggesting that more extensive nodal clearance may confer oncologic benefit in those undergoing sublobar resection. Researchers at Duke University analyzed 326,228 surgically treated NSCLC patients in the NCDB (2004–2014) and questioned the appropriateness of using 30-day postoperative

mortality as the primary metric for surgical quality assessment. Additional NCDB-based studies by Osarogiagbon et al. examined prognostic factors and the value of adjuvant therapy in incompletely resected NSCLC, while Mikell et al. demonstrated a survival benefit associated with postoperative radiotherapy in patients with N2-positive NSCLC. Although real-world studies on lung cancer surgery have begun to emerge in China, they remain relatively scarce and limited by smaller sample sizes.

Real-world research on lung cancer surgery is rapidly evolving from descriptive epidemiology toward hypothesis-driven investigations, aimed at addressing pressing clinical challenges and informing integrated treatment strategies. Therefore, large-scale real-world studies in China focusing on patients with surgically resected NSCLC are warranted. Leveraging big-data technologies to analyze comprehensive, real-world clinical data will enable comparative effectiveness research across multiple therapeutic modalities, facilitate the identification of optimal treatment algorithms, refine surgical quality control systems, and support the development of preoperative risk stratification models, ultimately advancing precision oncology for NSCLC patients.

2 Objective

The main objective for the NCC LungReal Phase 1 study is to establish a retrospective multicenter real-world database for patients who underwent surgery for lung cancer, and to develop an analytic framework to address clinical-relevant questions.

2.1 Database objective

Phase 1 of NCC LungReal study aims to create a real-world database with the following features:

- Including at least 12 medical centers.

- Including at least 60000 patients who underwent surgical resection for lung cancer between January 2014 and December 2021.

2.2 Data collection objective

Phase 1 study of NCC LungReal study aims to collect data on patient characteristics, treatment patterns, and outcomes. Efforts are taken to maximize completeness, comprehensiveness, and granularity of collected raw data.

2.3 Data processing objective

Due to limited computing and human resources, priorities of data processing were determined by the Study Committee. Resources in Phase 1 study are prioritized towards 1) baseline characteristics; 2) surgery information; 3) pathology report, with the required amount of human curation and computing resources in consideration. Part of data of low priority is anticipated to be processed in Phase 2 study.

2.3.1 Data with high priority

- Structured data that could be standardized into pre-defined terminologies and values.
- Surgery record.
- Pathology report. The Study Committee determined to put processing of pathology report to high priority based on the established prognostic value of multiple pathologic features.
- Clinical Tumor-Node-Metastasis staging.
- Pathologic Tumor-Node-Metastasis staging.
- Genetic testing.
- Overall survival.

2.3.2 Data with low priority

- Daily progress notes during hospital stay.
- Radiology report. The Study Committee determined to put processing of CT reports in low priority due to the very high complexity and very high inter-institutional and temporal variances of formatting and styling observed in radiology reports. Instead, the presence or absence of a certain type of radiologic examination will be extracted.

2.4 Data processing modules development objective

For unstructured raw data (pathology report, etc.), multiple modules integrating pattern extraction, machine learning, and natural language processing will be developed and optimized separately to adapt to raw data with different features and complexities.

2.5 Analysis objective

Qualified data were used for analyses to address the following objectives:

- The temporal trend of characteristics, treatment patterns, and outcomes for patients who received surgery for lung cancer.
- Overall survival in the whole study population, and in subgroups stratified by factors including temporal period of surgery, patient characteristics, histology, TNM stage, treatment pattern, and genetic alteration.
- Factors associated with the overall survival of patients.

3 Study design

3.1 Description of the study

Phase 1 study of NCC LungReal study is a retrospective, multicenter, electronic health record

(EHR)-based, real-world study. Figure 1 illustrates the study design.

Phase 1 study of NCC LungReal study enrolls patients who underwent surgical resection for lung cancer during the time period from January 2014 to December 2021.

3.2 Medical centers enrollment

3.2.1 Inclusion criteria

No limitation on type, level, location, or volume is set for enrollment of participating medical centers. All medical centers whose raw data are submitted to the National Cancer Information Database (NCID) that meet the predefined quality standards will be screened for study entry.

3.2.2 Exclusion criteria

Medical centers whose raw data submitted to NCID are incomplete, erroneous, or of low quality, as defined by meeting any of the following criteria, will be excluded:

(1) Proportion of cases that lacks front page of EHR, or cases whose front page is filled with unrelated text or garbled codes >40.0%.

(2) Proportion of cases that lack a pathology report of 60 or more Chinese characters, or cases whose pathology report is filled with unrelated text or garbled codes >40.0%.

(3) Proportion of cases that lack internal linkages of raw data from different sources >40.0%.

3.3 Patient enrollment

3.3.1 Inclusion criteria

Patients who were admitted to the enrolled participating medical centers between January 2014 to December 2021 and met any of the following criteria are eligible for study entry:

(1) Diagnosis of lung cancer and presence of lung surgery record.

(2) Diagnosis of lung cancer and presence of pathology report containing ≥60 Chinese characters.

(3) Diagnosis of lung cancer and presence of lung surgery in the data field of “operation” in the front page of EHR.

(4) Diagnosis of lung cancer and admitting department is (thoracic) surgery department.

“Diagnosis of lung cancer” requires any one of the following:

- Diagnoses on the front page of EHR include typical Chinese terminology for “lung cancer” and/or “lung malignancy” defined by the Study Committee.
- ICD-9 code on the front page of the medical record includes “162.”
- ICD-10 code on the front page of the medical record includes “c34.” or “c39.9”.
- ICD-O-3 code on the front page of the medical record includes any of xxxx/3 code in the list of 2015 WHO classification of lung tumors.
- Name of the surgery included typical Chinese terminology for lung cancer surgery, determined by the Study Committee.
- Pathology diagnosis includes terminologies for “lung cancer” and/or “lung malignancy” defined by the Study Committee.

3.3.2 Exclusion criteria

Patients who meet any of the following criteria will be excluded from study entry:

(1) Lacking raw data from the front page of the EHR.

(2) Lacking a pathology report of the surgically resected specimen.

(3) Lacking information required to link raw data from different sources together.

3.4 Outcome measurement

Overall survival (OS) is the primary outcome measurement.

OS is defined as the time from surgery to death from any cause. Patients who are lost to follow-up, or alive at last contact will be censored at last contact.

4 Method

4.1 Raw data collection

The source of raw data of National Cancer Center LungReal Study is National Cancer Information Database (NCID). Raw data are collected from electronic health record (EHR) systems of participating medical centers, including electronic medical record (EMR), hospital information system (HIS), laboratory information management system (LIS), and pathology information system (PIS), de-identified and encrypted, and transferred to NCID.

Before being processed by the NCC LungReal study, a separate set of unique identifiers will be assigned to patients and medical centers according to different naming rules from those of NCID to further reduce the risk of identity breaches.

4.2 Evaluation of raw data

Raw data is evaluated for completeness, integrity, and internal linkage. When a medical center is excluded according to the exclusion criteria, raw data from that medical center will be deleted. The Study Committee retains no copy of the raw data of an excluded medical center.

4.3 Evaluation of patient enrollment

Patient enrollment will be evaluated for accuracy and coverage manually.

4.3.1 Accuracy of patient enrollment

The accuracy of patient enrollment is defined as the proportion of patients enrolled that were confirmed by a human check.

As the lack of sex, age, and poor quality of internal linkage can be easily checked automatically, human resources are prioritized towards checking the presence of pathology reports for surgical specimens and the diagnosis of lung cancer. Trained data managers will check the raw text of the pathology report of enrolled patients to determine the presence of pathology reports for surgical specimens and the diagnosis of lung cancer. A total of 12,000 cases will be stratified-sampled from the included medical centers for accurate enrollment.

4.3.2 Coverage of patient enrollment

Patient enrollment coverage is defined as the proportion of patients from a human-curated external list who satisfy the enrollment criteria and were included in this study.

An external human-curated patient list of 8000 will be generated from designated medical center (s). Comparison against the external patient list may require identifying information and will be conducted exclusively by authorized data managers within a secure data center.

4.4 Data processing

Data processing aims to obtain standardized and structured values from structured and unstructured raw data. Numerous data processing modules will be developed to adapt to data with different types, formats, complexity, and processing difficulty.

4.4.1 Processing of structured raw data

Structured data with fixed values and format, including demographics information (age, sex, blood

type, etc.), dates, laboratory test results, peri-operative prescriptions, immunohistochemistry results, and genetic testing will be standardized into given allowable values according to dictionaries and transformation rules defined by the Study Committee.

4.4.2 Processing of pathology report

Human and computing resources will be prioritized towards the processing of pathology reports in Phase 1 study. According to previous experience working with pathology reports, pathology report is unstructured data in the form of natural language that could be digested to logical combination of medical terminologies. Some parts of a pathology report are structured data (e.g., lymph node examination, immunohistochemistry).

Briefly, the text of a pathology report will first be sectioned by a sectioning module into "gross description", "microscopic description of primary tumor", "examination of lymph node", "IHC results", and "genetic testing results". Text of "gross description" and "microscopic description of primary tumor" will be processed by two separate processing modules integrating pattern extraction, machine learning, and natural language processing. Text in the sections "examination of lymph node" and "IHC results" will be processed according to methods for processing structured data. Text in the section "genetic testing results" will be handed to the modules processing genetic testing results.

To achieve high precision and accuracy of processing of pathology reports, a Pathology Reference Set of structured pathology reports with a designed amount of 5000 cases will first be curated manually by certificated thoracic oncologists and data managers. This Pathology Reference Set will include all output data elements from pathology report and will serve as the gold standard to evaluate the performance of data processing.

4.4.3 Processing of other unstructured data

Unstructured text from the chief complaint, present history, past medical history, personal history, and family history will be processed separately. Modules integrating pattern extraction, machine learning, and natural language processing will be developed separately to adopt for different features of raw text.

4.4.4 Processing of surgery name

According to prior experience in processing surgery names, the total number of surgery names will be large but finite, forming a typical long-tail distribution. Surgery names will be manually curated independently by two board-certified thoracic surgeons, with complete concordance between them. Discordance will be determined by discussion with the Study Committee.

4.5 Quality evaluation of processed data

Quality evaluation of processed data is composed of rounds of preliminary quality tests and a finalized quality evaluation at the end of Phase 1 study.

4.5.1 Quality evaluation of processed data from structured data

According to prior experience in structured data, high accuracy of modules processing structured raw data is expected. Therefore, a relatively small amount (500 to 1000) of processed data will be stratified-sampled from enrolled medical centers and manually checked against raw data by trained data managers.

4.5.2 Quality evaluation of processed data from pathology data

Due to highly structured format, a limited number of processed lymph node examination and IHC

results will be selected using stratified sampling and manually verified against raw data by trained data managers.

For data elements processed from unstructured pathology reports, finalized quality evaluation includes 1) automatic comparison against Pathology Reference Set; 2) manual check of processed data against raw pathology reports stratified-sampled from participating centers conducted by trained data managers. The total number of cases, including those in the Pathology Reference Set, is expected to reach at least 12,000.

4.5.3 Quality evaluation of processed data from other unstructured data

According to prior experience in other unstructured data (chief complaint, present history, past medical history, personal history, and family history), the complexity of text and the technical difficulty of processing are expected to be manageable. A relatively small subset of processed data (500 to 1000 records) will be selected using stratified sampling from participating centers and manually verified against the raw data by trained data managers.

4.6 Follow-up and outcome

Follow-up and outcome data for included patients will be collected from multiple sources and harmonized by the Study Committee.

- Patients' visits or re-admissions to participating centers will be exported from NCID.
- Results of routine follow-up contacts through telephone calls performed by participating medical centers.
- National Cancer Center and Chinese Center for Disease Control and Prevention have established a cooperation framework, namely National Cancer Data Linkage (NCDL) Platform of

China [China CDC Wkly.2022;4(13):271-275.]. Death events collected by China CDC will be linked to NCC through NCDL and harmonized.

- The Study Committee plans to perform active follow-up. Due to the large number of enrolled patients, a relatively long interval (every 3 to 4 years) between each round of active follow-up is expected.

4.7 Timeline of Phase 1 study

Phase 1 study is scheduled to start between October and December 2019. Patient enrollment and data collection are expected to close on August 31, 2022. Development of data processing modules is anticipated to begin in December 2021, following the collection of the first batch of raw data. Finalized data quality evaluation for Phase 1 study is expected to be completed between August 2023 and January 2024.

5 Study governance

5.1 Administrative structure

National Cancer Center LungReal Study is sponsored by the National Cancer Center. The Principal Investigator Institution is the National Cancer Center/Cancer Hospital, Chinese Academy of Medical Sciences.

The Study Committee is responsible for drafting study protocols and designing Case Report Forms, General Data Models, and Data Element Book. The Study Committee is responsible for supervising the conduct of this study. The Study Committee is responsible for organizing the publication of study-related research articles or reports.

The National Cancer Center is responsible for the distribution, signing, and collection of relevant

forms and documents for this study.

5.2 Governance of data

The evaluation and processing of raw data, the development of processing modules, the evaluation of processed data, and the subsequent statistical analyses are conducted in a secure data center in National Cancer Center.

Access to hardware, software, raw data, processing rules, processing modules, processed data, and/or analytic results should be applied by submitting written application to Study Committee and the administrative authority of NCID.

Export of analytic results, tables, and figures could only be conducted with written approval from both the Study Committee and the administrative authority of NCID. Export of raw data, processed data, identifiable information, processing rules, and processing modules to outside of the secured data center is prohibited.

6 Ethics consideration

6.1 Compliance with laws and regulations

The design, conduction and reporting of this study comply with the ICH guideline for Good Clinical Practice, the ethical principles of the Helsinki Declaration, and local laws and regulations.

6.2 Informed consent

This study is a retrospective, non-interventional study using de-identified information in all analyses. According to the guidelines of the Council of International Medical Science Organizations (CIOMS), informed consent could be waived with the approval of the Ethics Committee. This study will apply for a waiver of informed consent.

6.3 Confidentiality standards

According to local laws and regulations, all identifiable information will be protected and anonymized into unreadable identifiers that cannot identify specific individuals.

The Study Committee maintains confidentiality standards by coding each patient and each medical center enrolled through the assignment of a unique identification id or code.

6.4 Change to study protocol

Any change, modification, or addition to the study protocol can only be applied in written form, and the revised protocol must be approved by the Institutional Review Board/Ethics Committee.
