# Supplementary material for: Bridging the lung cancer screening eligibility gap: evaluation of guideline applicability in asymptomatic patients
Source: Mil Med Res. 2026 Apr 15;13(1):100020. doi: 10.1016/j.mmr.2026.100020 (PMC13127149; doi:10.1016/j.mmr.2026.100020)
Supplement: Supplementary file 2 — Supplementary material [file mmc2.pdf]

Table S1 List of the international classification of diseases, tenth revision (ICD 10) codes

Comorbidity	ICD 10
Chronic respiratory diseases	D86−D86.2, D86.9, J41−J65.0, J84−J84.9, J92.0−J92.0
Hypertension	I10
Diabetes	E08−E08.11, E08.3−E08.9, E10−E10.11, E10.3−E11.1, E11.3−E12.1, E12.3−E13.11, E13.3−E14.1, E14.3−E14.9
Coronary heart disease	I20−I25.9

Table S2 Asymptomatic patients eligible and ineligible to lung cancer screening according to the USPSTF criteria in 2013 and 2021 [*n* (%)]

Screening eligibility criteria	USPSTF 2021 criteria	USPSTF 2013 criteria
Overall criteria		
Eligible	8985 (8.8)	6483 (6.3)
Ineligible	93,570 (91.2)	96,582 (93.7)
Age criteria		
Eligibility: 55–80 years old in 2013; 50–80 years old in 2021	81,422 (77.0)	66,914 (63.3)
Ineligibility: younger (<55 years old in 2013; <50 years old in 2021)	23,776 (22.5)	38,284 (36.2)
Ineligibility: older (>80 years old)	524 (0.5)	524 (0.5)
Smoking pack-year criteria		
Eligibility (>30 pack-years in 2013; >20 pack-years in 2021)	10,056 (9.9)	7921 (7.8)
Ineligibility (Non-smokers or smokers <30 pack-years in 2013; Non-smokers or smokers <20 pack-years in 2021)	91,928 (90.1)	94,063 (92.2)
Quit-year criteria		
Eligibility (≤15 years)	3133 (80.4)	3133 (80.4)
Ineligibility (>15 years)	763 (19.6)	763 (19.6)

Subgroup totals may not sum to the overall total because of missing values in some variables in the real-world database. USPSTF. US Preventive Services Task Force

Table S3 Joinpoint analysis of eligibility to lung cancer screening

Characteristics	Trend 1	APC [% (95% CI)]	Trend 2	APC [% (95% CI)]	AAPC [% (95% CI)]
USPSTF 2021 criteria					
All criteria	2014–2016	−12.2* (−18.3 to −5.6)	2016–2021	−19.4* (−24.7 to −16.6)	−17.4* (−19.1 to −15.9)
Age criteria	2014–2016	0.0 (−1.2 to 1.0)	2016–2021	−2.3* (−2.9 to −2.0)	−1.6* (−1.9 to −1.4)
Smoking pack-year criteria	2014–2021	−18.2* (−21.6 to −14.7)	-	-	−18.2* (−21.6 to −14.7)
Quit-year criteria	2014–2021	−0.3 (−1.5 to 0.9)	-	-	−0.3 (−1.5 to 0.9)
Screening ineligibility at stage Ia					
All criteria	2014–2016	6.3* (1.6–12.2)	2016–2021	2.0 (−3.7 to 5.0)	3.2* (1.6–4.6)
Age criteria (<50 years)	2014–2016	10.0* (4.2–16.7)	2016–2021	3.8 (−2.1, 6.4)	5.5* (3.7–7.0)
Smoking pack-year criteria	2014–2016	5.9* (1.0–12.3)	2016–2021	1.8 (−4.3 to 5.3)	3.0* (1.2–4.5)
Quit-year criteria	2014–2021	5.1* (2.0–8.4)	-	-	5.1* (2.0–8.4)
Sex					
Male	2014–2016	−7.0 (−14.9 to 2.3)	2016–2021	−16.1* (−23.8 to −12.7)	−13.6* (−16.0 to −11.6)
Female	2014–2021	−28.8* (−33.3 to −24.1)	-	-	−28.8* (−33.3 to −24.1)
Smoking status					
Current smokers	2014–2021	−0.7 (−2.0 to 0.6)	-	-	−0.7 (−2.0 to 0.6)
Former smokers	2014–2021	−1.5 (−4.5 to 1.7)	-	-	−1.5 (−4.5 to 1.7)

*The APC/AAPC is significantly different from zero (*P*<0.05). All models allowed a maximum of 1 joinpoint, up to 2 linear segments given the 8-year observation period. Trend 1 and Trend 2 denote the first and second trend segments, respectively. For analyses on screening ineligibility at stage Ia, asymptomatic patients aged >80 years were not included due to the insufficient sample size. "-" indicates that the corresponding estimate is not available, because no joinpoint was detected over the study period and the trend was modeled as a single segment. AAPC. Average annual percentage change; APC. Annual percentage change; CI. Confidence interval; USPSTF. US Preventive Services Task Force

Table S4 Characteristics of asymptomatic patients' ineligible to lung cancer screening stratified by sex per the USPSTF 2021 criteria [*n* (%)]

**Characteristics**	**Male (*n*=31,760)**	**Female (*n*=61,810)**	***P*-value**
Age			<0.001
<50 years	6764 (21.4)	17,012 (27.7)	
50–80 years	24,553 (77.6)	44,269 (72.0)	
>80 years	314 (1.0)	210 (0.3)	
Smoking status and pack years			<0.001
Non-smokers	27,014 (86.3)	61,148 (99.2)	
Smokers with <20 pack years	3326 (10.6)	440 (0.7)	
Smokers with 20–30 pack years	462 (1.5)	13 (0.0)	
Smokers with >30 pack years	486 (1.6)	13 (0.0)	
Smoking quit years for former smokers			<0.001
≤15 years	918 (56.0)	114 (73.6)	
>15 years	722 (44.0)	41 (26.5)	
Residence			0.996
Eastern region	29,484 (92.8)	57,380 (92.8)	
Central and western regions	2276 (7.2)	4430 (7.2)	
Family history of lung cancer in first-degree relatives			<0.001
No	30,443 (95.9)	58,288 (94.3)	
Yes	1317 (4.2)	3522 (5.7)	
Comorbidity			
Any comorbidity			<0.001
No	26,155 (82.4)	51,971 (84.1)	
Yes	5605 (17.7)	9839 (15.9)	
Chronic respiratory diseases			0.060
No	31,395 (98.9)	61,182 (99.0)	
Yes	365 (1.2)	628 (1.0)	
Hypertension			<0.001
No	27,474 (86.5)	54,061 (87.5)	
Yes	4286 (13.5)	7749 (12.5)	
Diabetes			<0.001
No	29,873 (94.1)	58,780 (95.1)	
Yes	1887 (5.9)	3030 (4.9)	
Coronary heart disease			0.011
No	31,050 (97.8)	60,582 (98.0)	

Characteristics	Male (*n*=31,760)	Female (*n*=61,810)	*P*-value
Yes	710 (2.2)	1228 (2.0)	
Medical insurance status			<0.001
No insurance coverage	10,058 (37.6)	16,624 (31.4)	
Have insurance coverage	16,723 (62.4)	36,340 (68.6)	
Lung cancer diagnosis			
Stage at diagnosis			<0.001
Carcinoma in situ	1437 (6.7)	4285 (10.1)	
Ia	14,675 (68.7)	32,343 (76.3)	
Ib	1440 (6.7)	1612 (3.8)	
IIa	520 (2.4)	397 (0.9)	
IIb	1220 (5.7)	1165 (2.8)	
IIIa	1899 (8.9)	2516 (5.9)	
IIIb	177 (0.8)	92 (0.2)	
IV	5 (0.0)	7 (0.0)	
Histological subtype			<0.001
Minimally invasive adenocarcinoma	3127 (11.7)	9323 (17.5)	
Adenocarcinoma	18,501 (69.2)	38,739 (72.5)	
Squamous carcinoma	2508 (9.4)	334 (0.6)	
Other NSCLC	2218 (8.3)	4896 (9.2)	
Small-cell lung cancer	368 (1.4)	129 (0.2)	
Mortality			<0.001
Overall death	1740 (5.5)	1405 (2.3)	
Alive or loss to follow-up	30,020 (94.5)	60,405 (97.7)	

Subgroup totals may not sum to the overall total because of missing values in some variables in the real-world database. NSCLC. Non-small cell lung cancer; USPSTF. US Preventive Services Task Force

Table S5 The distribution of original values and values after imputation [*n* (%)]

**Characteristic**	**Missing values**	**Distribution of original values**	**Distribution after imputation**
Age	544 (0.1)		
<50 years		23,776 (22.5)	23,910 (22.5)
50–80 years		81,422 (77.0)	81,832 (77.0)
>80 years		524 (0.5)	524 (0.5)
Sex	1 (0.0)		
Male		43,423 (40.9)	43,424 (40.9)
Female		62,842 (59.1)	62,842 (59.1)
Smoking status	1522 (1.4)		
Non-smokers		88,162 (84.2)	89,029 (83.8)
Former smokers		3933 (3.8)	4520 (4.3)
Current smokers		12,649 (12.1)	12,717 (12.0)
Smoking pack years	4282 (4.0)		
Smokers with <20 pack years		3766 (27.3)	4475 (26.0)
Smokers with 20–30 pack years		2135 (15.5)	2598 (15.1)
Smokers with >30 pack years		7921 (57.3)	10,164 (59.0)
Smoking quit years for former smokers	37 (0.0)		
≤15 years		763 (19.6)	908 (20.1)
>15 years		3133 (80.4)	3612 (79.9)
Medical insurance status	16,075 (15.1)		
No		27,980 (31.0)	32,408 (30.5)
Yes		62,211 (69.0)	73,858 (69.5)

Subgroup totals may not sum to the overall total because of missing values in some variables in the real-world database

Table S6 Sensitive analysis of mortality risk accounting for screening utilization

**Screening eligibility and utilization**	***HR*** **(95% CI)**	***P*****-value**
Overall		
Eligible, without screening utilization	Reference	
Eligible, with screening utilization	0.58 (0.30–1.16)	0.122
Screening ineligible	0.62 (0.49–0.77)	<0.001
Stage I		
Eligible, without screening utilization	Reference	
Eligible, with screening utilization	0.76 (0.27–2.13)	0.596
Screening ineligible	0.68 (0.47–1.00)	0.047
Stage II		
Eligible, without screening utilization	Reference	
Eligible, with screening utilization	-	0.988
Screening ineligible	0.85 (0.44 −1.66)	0.638
Stage III		
Eligible, without screening utilization	Reference	
Eligible, with screening utilization	-	0.981
Screening ineligible	0.93 (0.57 −1.54)	0.787

HRs were adjusted for sex, comorbidity, family history of lung cancer in first-degree relatives, and insurance coverage status. "-" means that *HR* with corresponding 95% CI could not be reliably estimated due to the insufficient number of mortality cases in the subgroups of screening-eligible individuals with screening utilization at stages II and III. CI. Confidence interval

Table S7 Sensitive analysis of mortality risk by defining screening as chest CT performed >12 months before surgery

**Screening eligibility and utilization**	***HR* (95% CI)**	***P*-value**
Overall		
Eligible, without screening utilization	Reference	
Eligible, with screening utilization	0.68 (0.30–1.54)	0.352
Screening ineligible	0.63 (0.51–0.79)	<0.001
Stage I		
Eligible, without screening utilization	Reference	
Eligible, with screening utilization	0.73 (0.18–3.03)	0.663
Screening ineligible	0.69 (0.48–1.00)	0.050
Stage II		
Eligible, without screening utilization	Reference	
Eligible, with screening utilization	-	0.988
Screening ineligible	0.88 (0.45–1.72)	0.714
Stage III		
Eligible, without screening utilization	Reference	
Eligible, with screening utilization	-	0.979
Screening ineligible	0.96 (0.58–1.58)	0.858

HRs were adjusted for sex, comorbidity, family history of lung cancer in first-degree relatives, and insurance coverage status. "-" means that *HR* with corresponding 95% CI could not be reliably estimated due to the insufficient number of mortality cases in the subgroups of screening-eligible individuals with screening utilization at stages II and III. CI. Confidence interval

Table S8 Sensitive analysis of mortality risk by excluding those with pre-existing chronic respiratory diseases

**Screening eligibility and utilization**	***HR* (95% CI)**	***P*-value**
Overall		
Eligible, no screening utilization	Reference	
Eligible, screening utilization	0.53 (0.26–1.09)	0.083
Screening ineligible	0.63 (0.50–0.78)	<0.001
Stage I		
Eligible, no screening utilization	Reference	
Eligible, screening utilization	0.57 (0.18–1.86)	0.354
Screening ineligible	0.67 (0.46–0.98)	0.041
Stage II		
Eligible, no screening utilization	Reference	
Eligible, screening utilization	-	0.988
Screening ineligible	0.92 (0.47–1.79)	0.795
Stage III		
Eligible, no screening utilization	Reference	
Eligible, screening utilization	-	0.982
Screening ineligible	0.96 (0.57–1.62)	0.886

*HR*s were adjusted for age group, family history of lung cancer in first-degree relatives, and insurance coverage status. "-" means that *HR* with corresponding 95% CI could not be reliably estimated due to the insufficient number of mortality cases in the subgroups of screening-eligible individuals with screening utilization at stages II and III. CI. confidence interval

Table S9 Sensitivity analysis using fine-gray test for lung cancer mortality

**Screening eligibility**	***HR* (95% CI)**	***P*-value**
Overall		
Screening eligible	Reference	
Screening ineligible	0.58 (0.52–0.65)	<0.001
Stage I		
Screening eligible	Reference	
Screening ineligible	0.56 (0.46–0.69)	<0.001
Stage II		
Screening eligible	Reference	
Screening ineligible	1.20 (0.93–1.56)	0.162
Stage III		
Screening eligible	Reference	
Screening ineligible	0.82 (0.67–1.01)	0.059

*HR*s were adjusted for sex, comorbidity, family history of lung cancer in first-degree relatives, insurance coverage status, and residence

Table S10 Baseline characteristics of participants and stratified by screening eligibility based on 5 cities from NLCS [*n* (%)]

Baseline characteristics	Overall participants (*n* = 353,179)	USPSTF 2021 criteria	
		Screening ineligible (*n* = 314,208)	Screening eligible (*n* = 38,971)
Age			
<50 years	74,958 (21.2)	74,958 (23.9)	0 (0.0)
50–80 years	278,221 (78.8)	239,250 (76.1)	38,971 (100.0)
Smoking status and pack years			
Non-smokers	264,363 (74.9)	264,363 (84.1)	0 (0.0)
Smokers with <20 pack years	41,614 (11.8)	41,614 (13.2)	0 (0.0)
Smokers with ≥20 pack years	47,202 (13.4)	8231 (2.6)	38,971 (100.0)
Smoking quit years for former smokers			
≤15 years	3037 (17.7)	3037 (25.4)	0 (0.0)
>15 years	14,082 (82.3)	8928 (74.6)	5154 (100.0)
Sex			
Male	146,815 (41.6)	111,496 (35.5)	35,319 (90.6)
Female	206,364 (58.4)	202,712 (64.5)	3652 (9.4)
Family history of lung cancer in first-degree relatives			
No	308,747 (87.4)	279,904 (89.1)	28,843 (74.0)
Yes	44,432 (12.6)	34,304 (10.9)	10,128 (26.0)
Comorbidity			
Hypertension			
No	256,266 (72.6)	233,122 (74.2)	23,144 (59.4)
Yes	96,913 (27.4)	81,086 (25.8)	15,827 (40.6)
Chronic respiratory diseases			
No	283,230 (80.2)	260,117 (82.8)	23,113 (59.3)
Yes	69,949 (19.8)	54,091 (17.2)	15,858 (40.7)
Diabetes			
No	317,919 (90.0)	285,211 (90.8)	32,708 (83.9)
Yes	35,260 (10.0)	28,997 (9.2)	6263 (16.1)

Not all percentages add up to 100% because of rounding. Five cities (Beijing, Shenyang, Hangzhou, Hefei, and Ningbo) out of the 12 participating cities (the remaining cities were Changsha, Zhengzhou, Zhumadian, Anyang, Quzhou, Xuzhou, Nanjing) in NLCS were selected for external validation, based on the inclusion of their local cancer registries in the cancer incidence in 5 continents (CI5) database. Subgroup totals may not sum to the overall total because of missing values in some variables in the real-world database. NLCS. National lung cancer screening cohort; USPSTF. US Preventive Services Task Force

Table S11 Lung cancer incidence stratified by screening eligibility per the USPSTF 2021 criteria based on 5 cities from NLCS [*n* (%)]

Lung cancer incidence	USPSTF 2021 criteria		Age criteria		Pack-year criteria		Quit-year criteria§	
	Screening ineligible	Screening eligible	Screening ineligible (<50 years)	Screening eligible (≥50 and ≤80 years)	Screening ineligible (Non-smokers or smokers with <20 pack years)	Screening eligible (Smokers with ≥20 pack years)	Screening ineligible (>15 years)	Screening eligible (≤15 years)
Total	1409 (0.5)	441 (1.1)	130 (0.2)	1720 (0.6)	1392 (0.5)	458 (1.0)	15 (0.5)	85 (0.6)
Stage at diagnosis*								
0–I	732 (68.0)	143 (45.4)	89 (83.2)	786 (61.2)	725 (68.3)	150 (45.6)	4 (33.3)	36 (60.0)
II	55 (5.1)	24 (7.6)	6 (5.6)	73 (5.7)	53 (5.0)	26 (7.9)	0 (0.0)	1 (1.7)
III	84 (7.8)	63 (20.0)	2 (1.9)	145 (11.3)	84 (7.9)	63 (19.2)	3 (25.0)	9 (15.0)
IV	205 (19.1)	85 (27.0)	10 (9.4)	280 (21.8)	200 (18.8)	90 (27.4)	5 (41.7)	14 (23.3)

*Available data on stage at diagnosis were presented. Not all percentages add up to 100% because of rounding. §There were no significant statistical differences ($P>0.05$) in distributions of lung cancer stage between former-smokers with ≤15 quit years and those >15 quit years. Five cities (Shenyang, Beijing, Hangzhou, Hefei, and Ningbo) out of the 12 participating cities (the remaining cities were Changsha, Zhengzhou, Zhumadian, Anyang, Quzhou, Xuzhou, and Nanjing) in NLCS were selected for external validation, based on the inclusion of their local cancer registries in the cancer incidence in 5 continents (CI5) database. Subgroup totals may not sum to the overall total because of missing values in some variables in the real-world database. NLCS. National Lung Cancer Screening; USPSTF. US Preventive Services Task Force

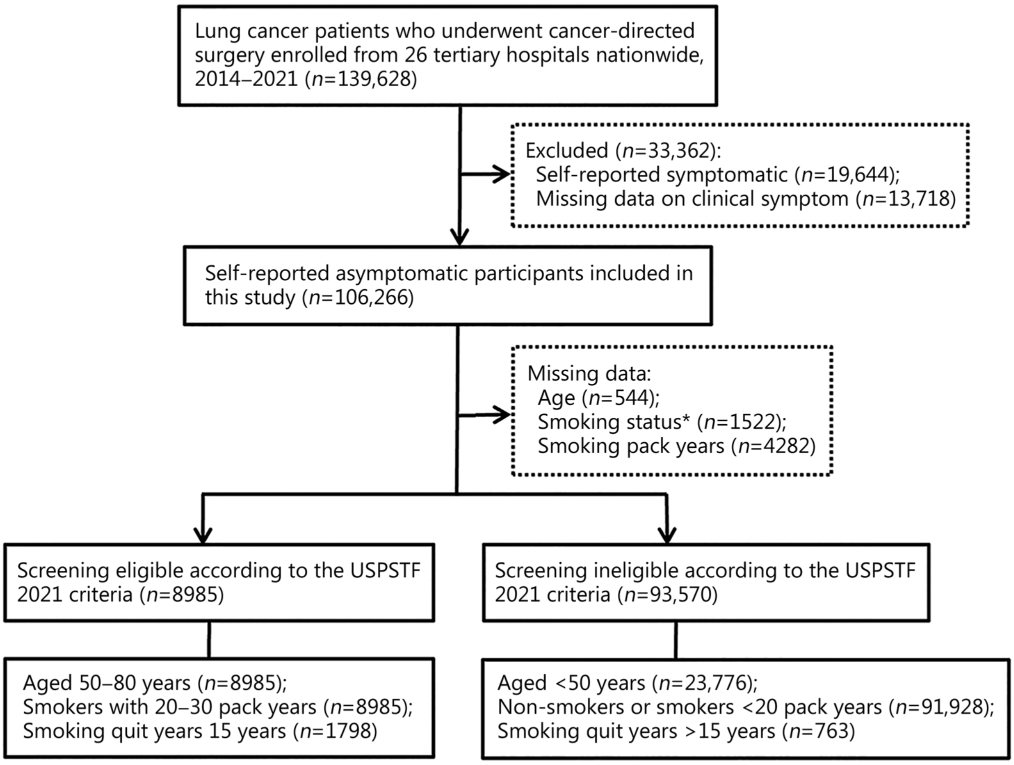

Fig. S1 Flow diagram of patient selection and exclusion criteria. *Smoking status: non-smokers; former smokers; current smokers

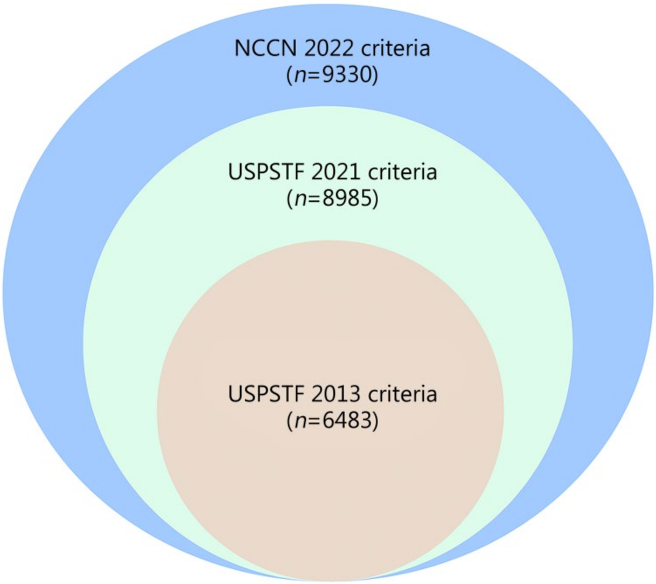

Fig. S2 The proportion of asymptomatic patients eligible to lung cancer screening according to the US Preventive Services Task Force (USPSTF) and the National Comprehensive Cancer Network (NCCN) criteria

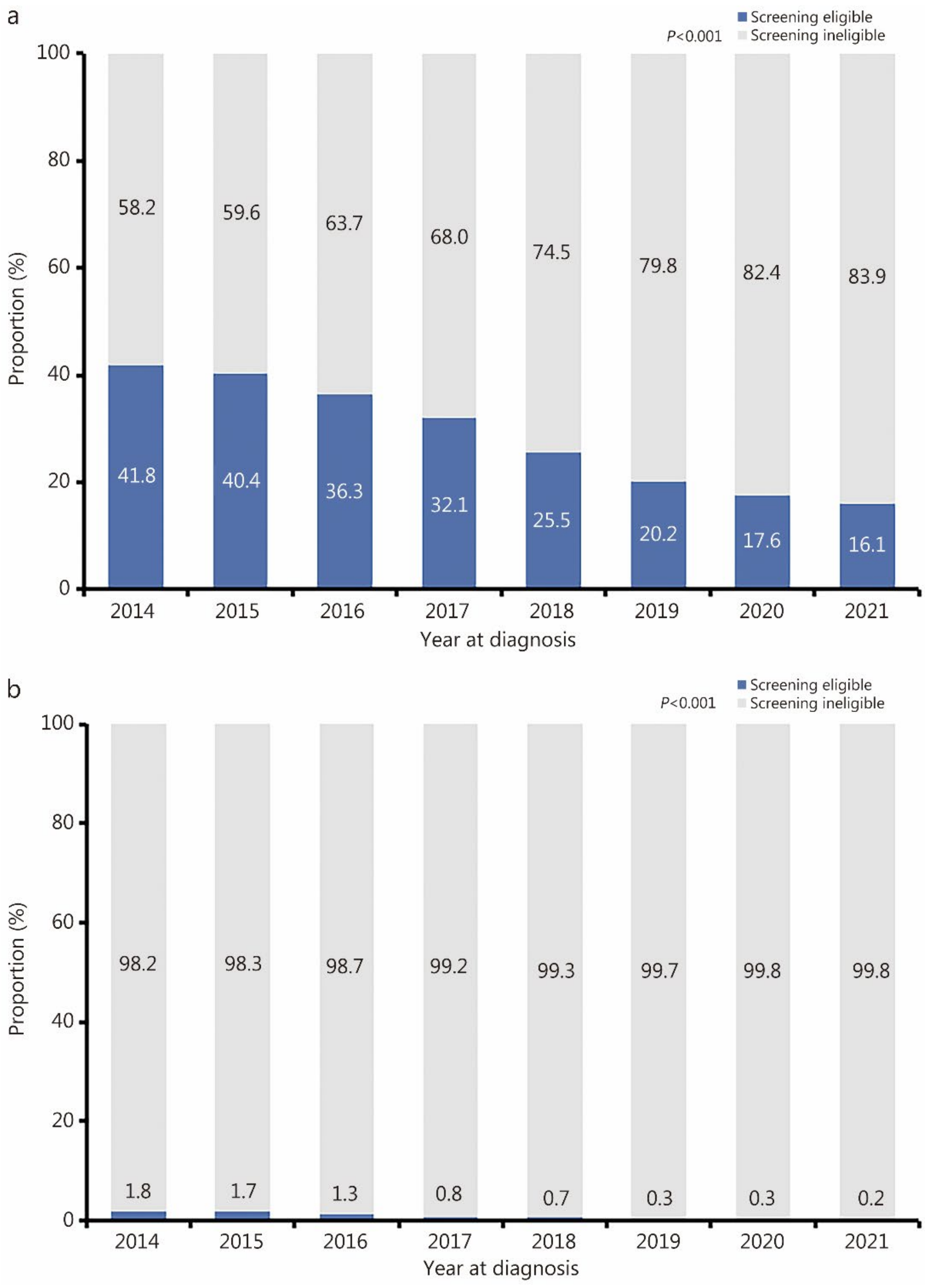

Fig. S3 Trends of eligibility to lung cancer screening among asymptomatic patients stratified by sex according to the US Preventive Services Task Force (USPSTF) 2021 criteria. **a** Males eligible to the USPSTF 2021 criteria. **b** Females eligible to the USPSTF 2021 criteria

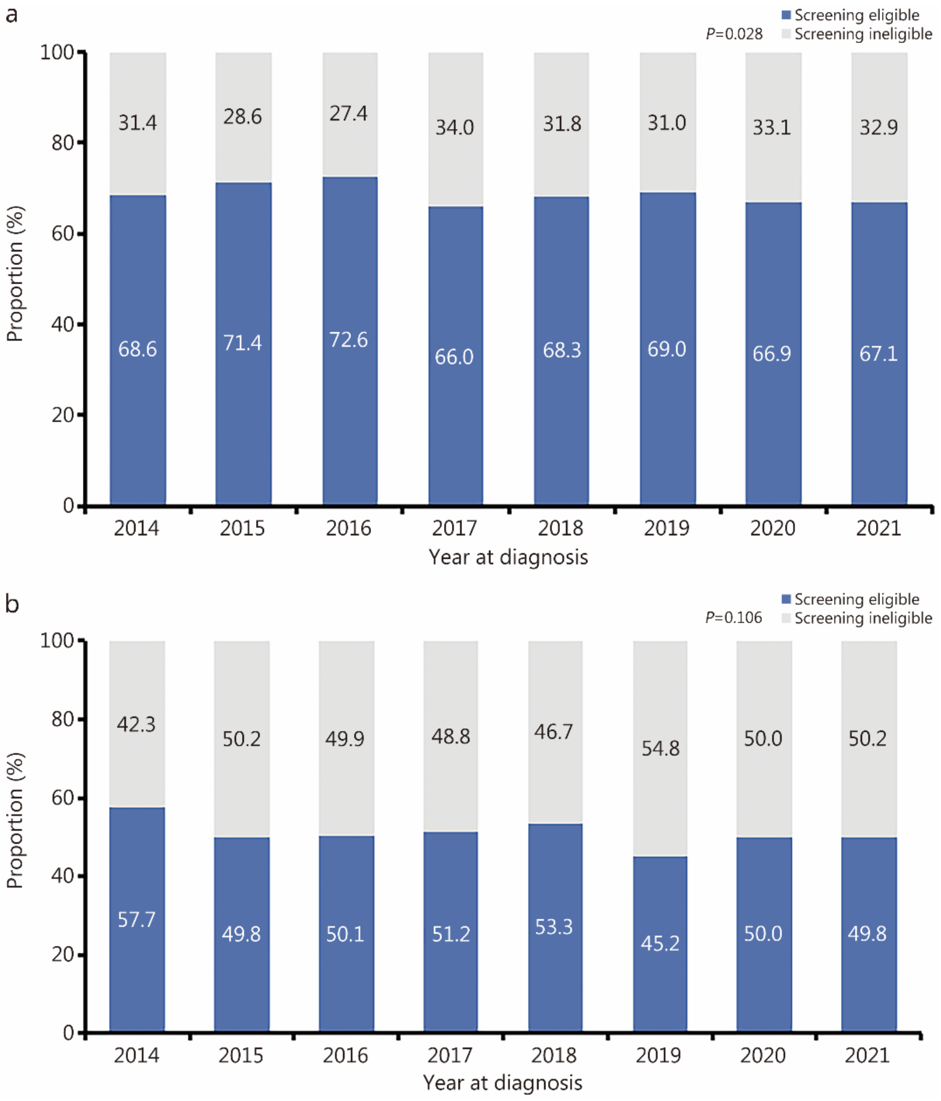

Fig. S4 Trends of eligibility to lung cancer screening among asymptomatic patients who currently or formerly smoked according to the US Preventive Services Task Force (USPSTF) 2021 criteria. **a** Current smoker. **b** Former smokers

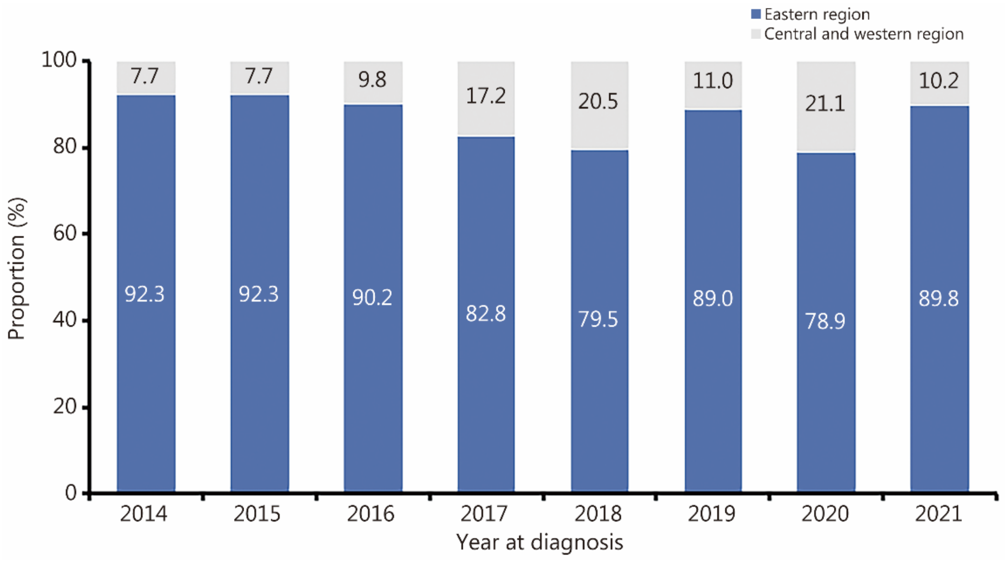

Fig. S5 Trends in the regional proportion among asymptomatic patients eligible for lung cancer screening according to the US Preventive Services Task Force (USPSTF) 2021 criteria. Eastern/Central and Western regions were classified based on the National Bureau of Statistics classification

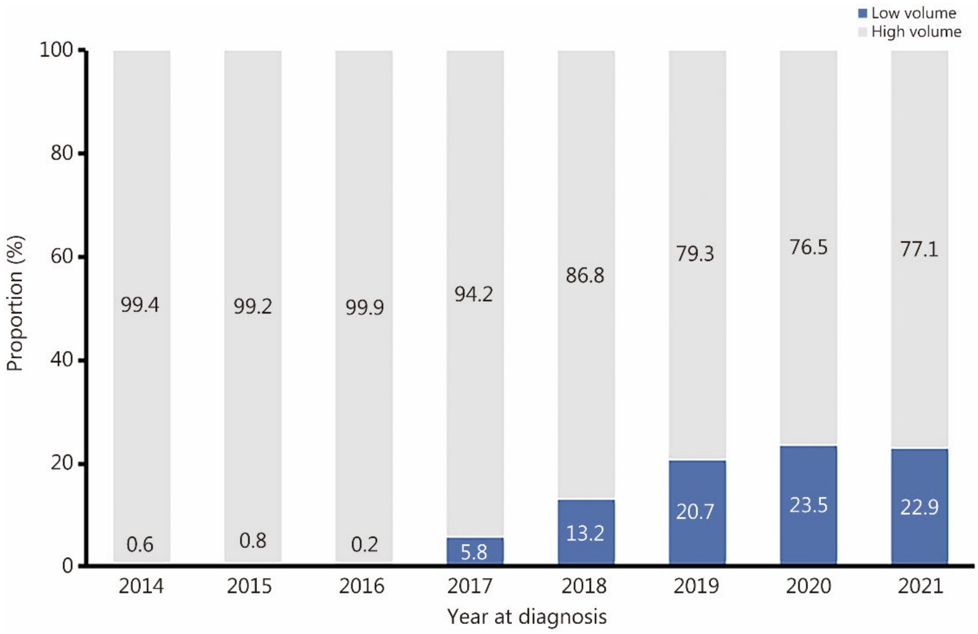

Fig. S6 Trends in the proportion of surgery volume among asymptomatic patients eligible for lung cancer screening according to the US Preventive Services Task Force (USPSTF) 2021 criteria. Low and high volume denote surgery volumes at or below, and above the third quartile across hospitals, respectively

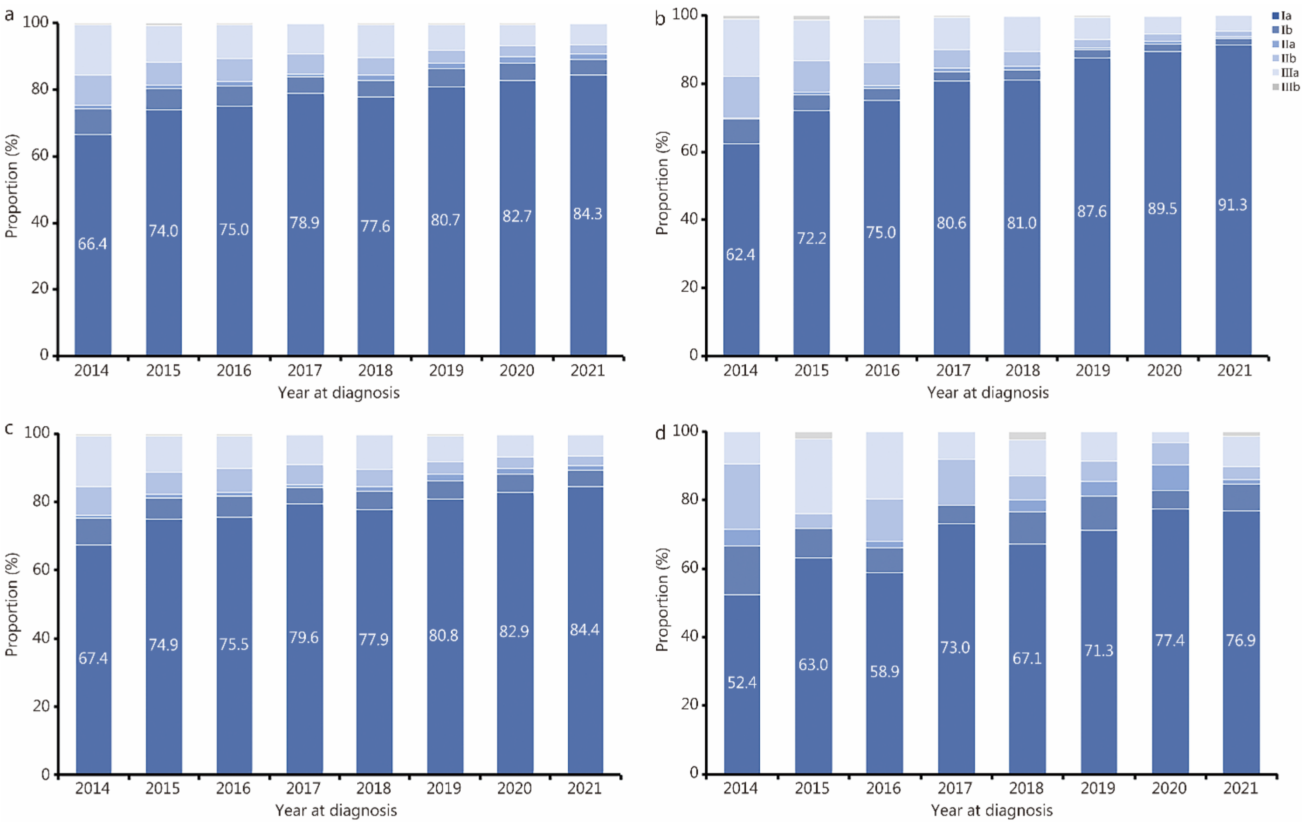

Fig. S7 Trends of stage at diagnosis among asymptomatic patients' ineligible to lung cancer screening according to the US Preventive Services Task Force (USPSTF) 2021 criteria. **a** Fail to fulfill the USPSTF 2021 criteria. **b** Fail to fulfill the screening criteria on age (<50 years). **c** Fail to fulfill the screening criteria on smoking pack years (non-smokers or smokers with <20 pack years). **d** Fail to fulfill the screening criteria on smoking quit years (quit years >15)

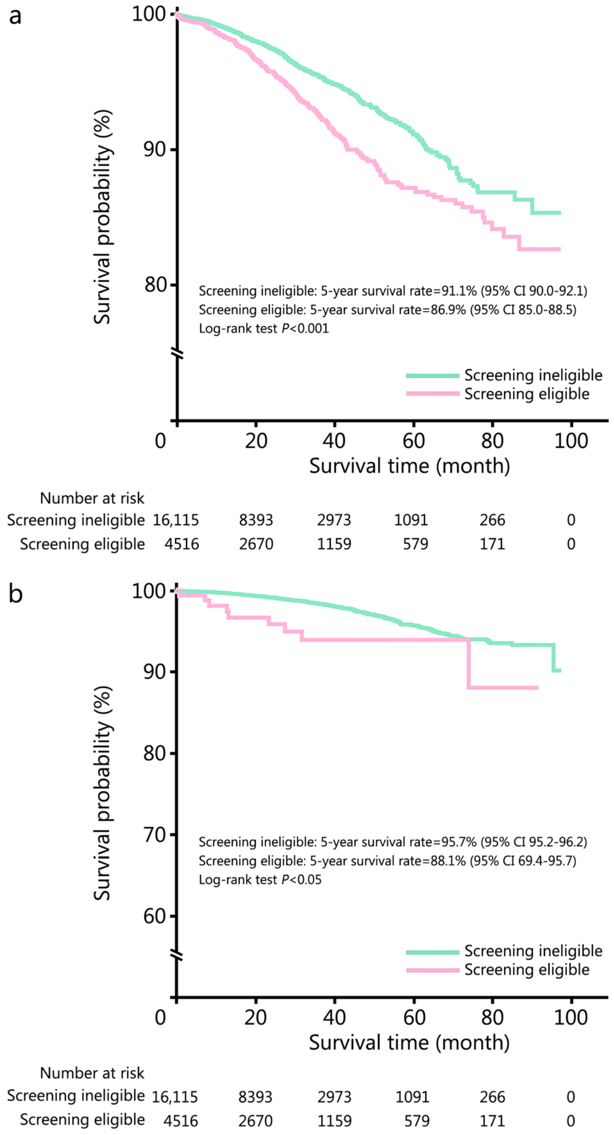

Fig. S8 Kaplan-Meier estimates among asymptomatic lung cancer patients at stage I according to the US Preventive Services Task Force 2021 criteria stratified by sex. **a** Males. **b** Females. CI. Confidence interval

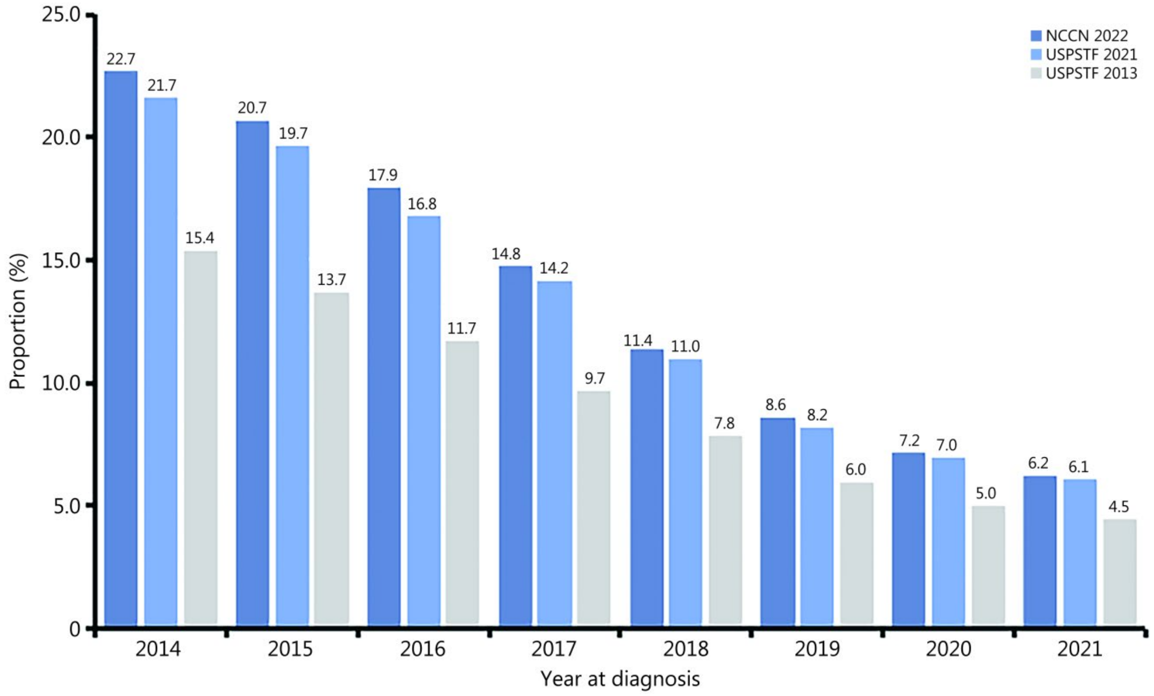

Fig. S9 The proportion of asymptomatic patients eligible to lung cancer screening according to different guidelines. Screening eligible: asymptomatic individuals who fulfill all the following criteria. NCCN 2022 criteria: 1) age criteria: aged ≥50 years; 2) smoking pack-year criteria: a smoking history of ≥20 pack-year smoking history. USPSTF 2021 criteria: 1) age criteria: aged 50–80 years; 2) smoking pack-year criteria: a smoking history of ≥20 pack-years for current or former smokers; 3) quit-year criteria: cessation ≤15 years for former smokers. USPSTF 2013 criteria: 1) age criteria: aged 55–80 years; 2) smoking pack-year criteria: a smoking history of ≥30 pack-years; 3) had quit smoking for ≤15 years for former-smokers. NCCN. National Comprehensive Cancer Network; USPSTF. US Preventive Services Task Force

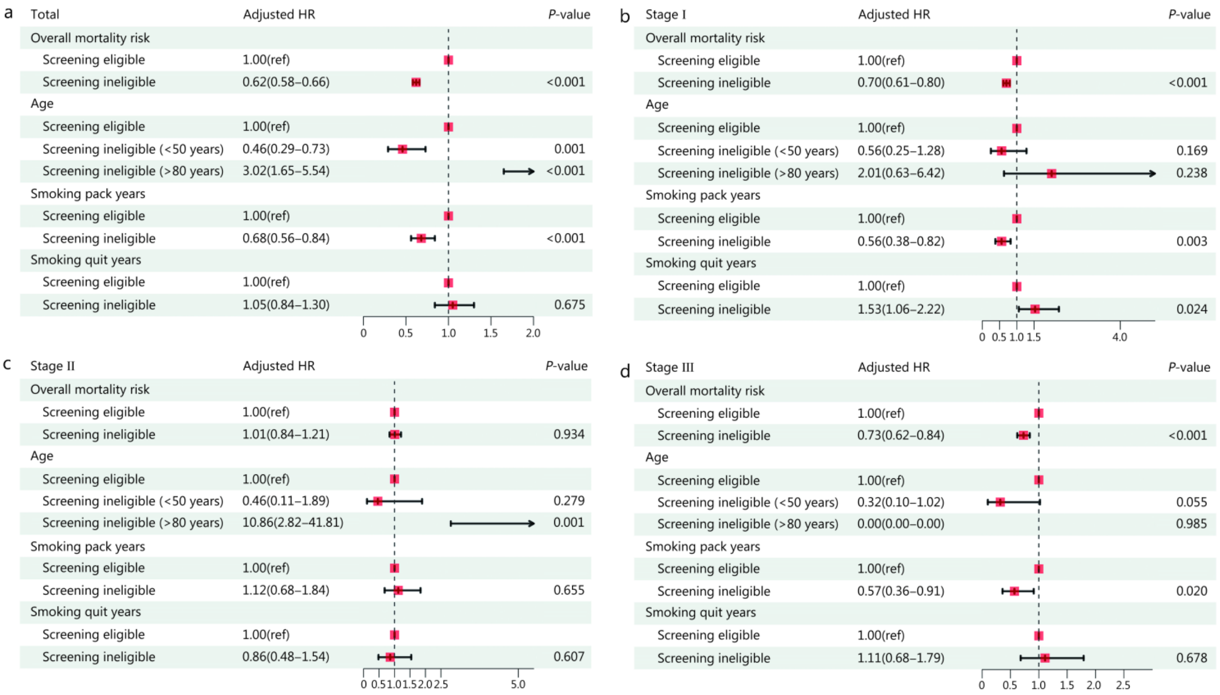

Fig. S10 Sensitive analysis of mortality risk by the US Preventive Services Task Force (USPSTF) 2021 eligibility after imputation. **a** Total asymptomatic patient. **b** Stage I. **c** Stage II. **d** Stage III. Hazard ratios (*HR*s) were adjusted for sex, comorbidity, family history of lung cancer in first-degree relatives, insurance coverage status, and residence after imputation. Adjusted *HR* for patients aged >80 years at stage III could not be estimated steadily due to the insufficient mortality cases
